# An integrated modelling framework from cells to organism based on a cohort of digital embryos

**DOI:** 10.1038/srep37438

**Published:** 2016-12-02

**Authors:** Paul Villoutreix, Julien Delile, Barbara Rizzi, Louise Duloquin, Thierry Savy, Paul Bourgine, René Doursat, Nadine Peyriéras

**Affiliations:** 1 BioEmergences Laboratory USR3695, CNRS, Université Paris-Saclay, 91198 Gif-sur-Yvette Cedex, France; 2 Complex Systems Institute Paris Île-de-France (ISC-PIF) UPS3611, CNRS, 113 rue Nationale, 75013 Paris, France

## Abstract

We conducted a quantitative comparison of developing sea urchin embryos based on the analysis of five digital specimens obtained by automatic processing of *in toto* 3D+ time image data. These measurements served the reconstruction of a prototypical cell lineage tree able to predict the spatiotemporal cellular organisation of a normal sea urchin blastula. The reconstruction was achieved by designing and tuning a multi-level probabilistic model that reproduced embryo-level dynamics from a small number of statistical parameters characterising cell proliferation, cell surface area and cell volume evolution along the cell lineage. Our resulting artificial prototype was embedded in 3D space by biomechanical agent-based modelling and simulation, which allowed a systematic exploration and optimisation of free parameters to fit the experimental data and test biological hypotheses. The spherical monolayered blastula and the spatial arrangement of its different cell types appeared tightly constrained by cell stiffness, cell-adhesion parameters and blastocoel turgor pressure.

Robust and reproducible animal embryonic development requires the coordination of a large number of cells. Yet, single-cell processes are inherently noisy and can lead to significant variations and heterogeneity within a priori homogeneous cell populations^1^,^2^,^3^. Recent advances in the quantitative live imaging of whole embryos^4^, including cell lineage “reconstruction”^5^,^6^,^7^ and gene expression “atlasing”^8^, mainly in the zebrafish, fruit fly, and nematode, provide an important path toward reconciling the two aspects of robustness and variability. The *in toto* maps at cellular resolution produced by these works allow deciphering the relationships between the single-cell features and the embryo-level dynamics underlying morphogenesis.

Sea urchin species are model organisms of choice in developmental biology. The structure and dynamics of the gene regulatory network (GRN) of *Strongylocentrotus purpuratus* have been extensively studied, leading to complete models of interactions between genes^9^,^10^,^11^. *Paracentrotus lividus*, on the other hand, is better suited for *in toto* imaging at the individual-cell resolution over long periods of time due to its transparency and robustness under experimental conditions^6^,^12^. We deliver here the first complete methodological framework for the predictive understanding of animal embryogenesis combining *in toto* 3D+ time imaging, statistical and mechanical modelling. We performed a fully automated reconstruction of digital specimens from live *P. lividus* embryos throughout the blastula stages to assess intra-individual variations and inter-individual differences at the level of groups of cells. Analysing the large amount of data generated by such tools requires novel methodological approaches^13^,^14^,^15^,^16^. We combined data organisation, multi-level probabilistic modelling and data fusion techniques, applied to measurable parameters, with spatially explicit biomechanical modelling^17^,^18^ to infer the remaining free parameters. This hybrid strategy led to a realistic prototypical simulation of the sea urchin lineage tree and developing embryo in 3D, directly comparable to empirical data. Ultimately, the systematic exploration of the model’s parameter space highlighted the developmental constraints of embryonic morphogenesis and its characteristic features such as the embryo shape and organisation of cell types.

## Results

### A cohort of digital embryos

**Figure 1:**
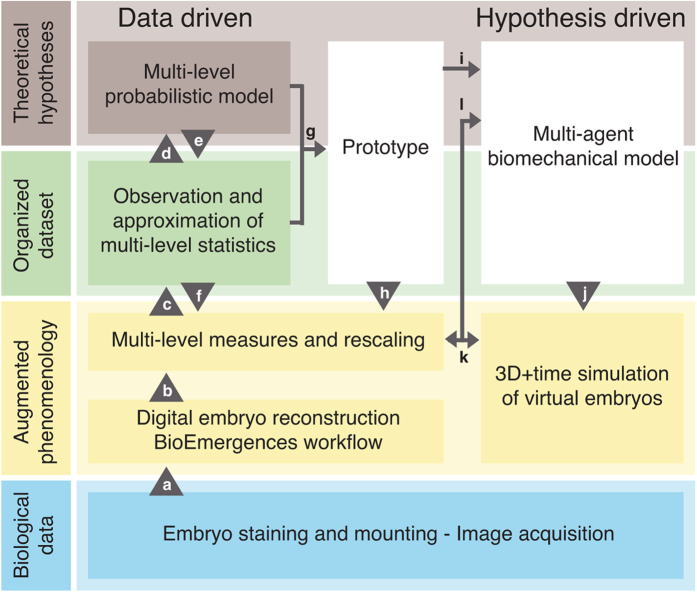
Methodological workflow. The technical content of each box is described in detail in the supplementary material. Bottom to top: increasing levels of abstraction, from raw data to theory and modelling. The concept of “augmented phenomenology” (second tier) represents the superposition of raw data and its reconstruction. Features extracted from the augmented phenomenology are combined into an organised dataset conveying maximum biological meaning and leading to the formulation of theoretical hypotheses. (**a** to **d**) Upward arrowheads indicate derivation from data, including reconstruction of digital specimens and statistical analysis leading to probabilistic models. (**e** and **f**, **h** and **j**) Downward arrowheads indicate prediction testing, whether analytically (**e**) or by simulation (**f**), (**h**) and (**j**). Horizontal arrow: (**g**) Aggregation step leading to the design of a “normal” prototype from measurable individual cell features across the five specimens. (**i**) The prototype is used as an input into the biomechanical model. (**k**) Bidirectional arrow indicating the quantitative comparison between model simulations and digital reconstruction. (**l**) Feedback loop tuning the parameter values of the biomechanical model as a function of realism.

**Figure 2:**
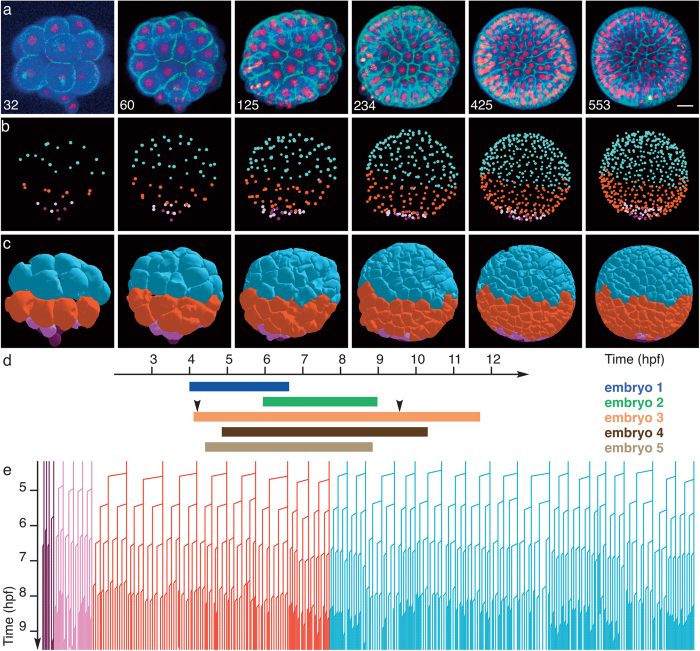
Reconstruction of digital sea urchins from 3D+ time *in vivo* and *in toto* imaging. (**a** to **c**) Raw and reconstructed data from one specimen (embryo 3) at different stages of development. Scale bar 20 μm. (**a**) Volume rendering of raw images (Amira software) from two-photon laser scanning microscopy. Total cell number indicated bottom left. (**b,c**) Reconstructed digital embryo, displayed with Mov-IT software. (**b**) Detected nuclear centres with colour code for cell types propagated along the lineage: mesomeres (Mes) in blue, macromeres (Mac) in red, large micromeres (LMic) in pink, and small micromeres (SMic) in purple. (**c**) Surface rendering of segmented cell membranes, colour code as in (**b**). (**d**) Temporal observation window for each specimen of the cohort, time scale in hours post-fertilisation (hpf) after temporal rescaling (details in Supplementary Fig. 2). Arrowheads for embryo 3 indicate the temporal window displayed in (**e**). (**e**) Flat representation of the reconstructed cell lineage of embryo 3 with the same cell type colour code as in (**b**), time scale in hpf.

Images of five live embryos developing from the 32-cell stage at 4–6 hours post-fertilisation (hpf) until the hatching blastula were acquired with two-photon microscopy and processed by our automated reconstruction workflow^5^,^6^ (Fig. 1a and Supplementary Table 1). Nuclear and membrane staining were obtained by RNA injection at the one-cell stage (Fig. 2a). This produced spatiotemporal sets of cell centres, segmented membrane shapes, and the complete cell lineage tree (Fig. 2b,c,e) via automated identification of cell filiation across consecutive time steps. Image acquisition lasted 3–8 consecutive hours with a constant time resolution of 2–5 min (Fig. 2d). Our visualisation interface Mov-IT^5^,^6^ helped validate and correct cell tracking, and manually label cells at the 32-cell stage according to their classification into four cell types with known distinct fates: mesomeres (Mes), macromeres (Mac), large micromeres (LMic) and small micromeres (SMic) (Supplementary Fig. 1b)^11^,^19^. Labels were propagated along the cell lineage (Supplementary Video 1). This data was suitable to investigate the parameters characterising cell behaviour, including cell displacements, cell divisions, cell volume, cell shape and cell contact changes.

### Temporal and spatial rescaling of embryo-level dynamics

**Figure 3:**
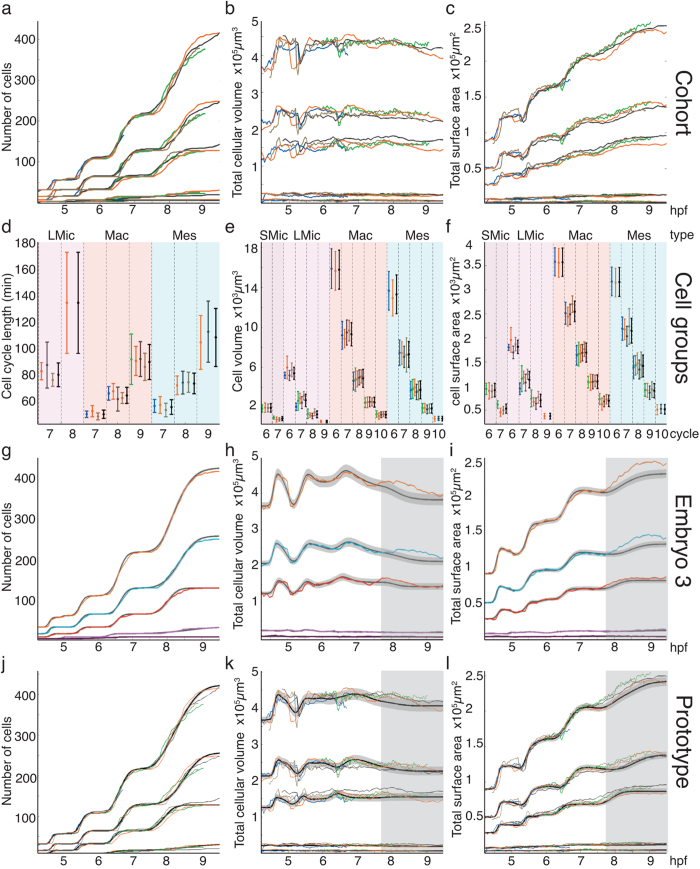
Multi-level statistics and probabilistic model for the cohort and its prototype. (**a** to **c**) Temporally rescaled macroscopic quantities (whole-embryo level) in each specimen, top groups of curves in each chart and each cell population (lower four groups of curves, top to bottom: Mes, Mac, LMic, SMic). Same embryo colour code as in Fig. 2d (details in Supplementary Fig. 2). (**d** to **f**) Statistics of individual cell features in each cell group clustered by common type and generation. Mean and standard deviation bars represent normal and log-normal approximations of the cell feature distributions. In black: statistics aggregated into a prototype (see Supplementary Figs 3 and 4). (**g** to **i**) Embryo-level features in one measured specimen (embryo 3) and in simulation based on the probabilistic model (see Supplementary Fig. 11 for details). In black: mean of 300 simulated cell lineages; in grey: their standard deviation; in colour: measured values (top curve: whole embryo, lower four curves: separate populations using the same cell type colours as Fig. 2b). Shaded areas: incomplete cycles, for which an accurate estimation was not possible. (**j** to **l**) Same as (**a** to **c**) with the prototype curves added in black and grey.

The quantitative inter-individual comparison of morphogenetic features was first measured at the global embryo level at each time step via the total number of cells *N*(*t*), total cellular volume *W*(*t*) and total cellular surface area *Z*(*t*). Because embryos appeared to grow at different speeds, a spatiotemporal rescaling of the corresponding curves was necessary to establish a baseline for inter-individual comparison and the assessment of variability (Fig. 3a–c), i.e. to provide comparable charts while preserving the overall shape of the functions. This rescaling consisted of an affine transformation in time and a linear transformation in space, tuned by parameters specific to each embryo (Supplementary Fig. 2c,f). After this preliminary step, the temporal evolution of the above three global measures was found to be highly reproducible between specimens of the cohort (Fig. 3a–c and Supplementary Fig. 2). Egg size and temperature fluctuations are potential factors explaining growth rate variation at the level of the embryo. Using this rescaling, we provided a correspondence between hpf and developmental stages (Supplementary Table 2).

### Coarse-grained statistical analysis of cell features along the cell lineage

We further investigated the possibility to relate the global similarity of embryos (macroscopic level of description) to individual cell features (microscopic level of description). To this aim, we examined how six features of individual cells were distributed along the cell lineage: cell cycle lengths *x*_*i*_(*t*), mitosis times *m*_*i*_(*t*) (Supplementary Fig. 1a), average cell volumes 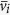
(*t*) and cell surface areas 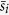
(*t*), average daughter/mother volume ratios *a*_*i*_(*t*) = 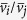 and daughter/mother surface area ratios *b*_*i*_(*t*) = 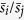. Because symmetries in the embryos, namely rotations around the animal-vegetal axis, and variations in single cell positions from one embryo to the other prevented the identification of individual cells across specimens, a “coarse-graining” transition to a mesoscopic and more generic level of description was required (Fig. 1c). To this aim, cells were clustered into groups *g* according to their type *k*_*i*_ = 1, …, 4 (Mes, Mac, LMic, SMic) and their generation rank *n*_*i*_ = 6, …, 10. Assuming that cells were indistinguishable within the same group, individual cell features were “exchangeable” sequences of random variables, hence obeyed a theorem stating that such sequences are mixtures of independent and identically distributed (i.i.d.) variables^20^. The above six cell features thus produced six distributions of individual cell features at the level of each group: *X*_*g*_, *M*_*g*_, 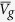, 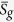, *A*_*g*_ and *B*_*g*_, which we set out to measure.

Due to limits on the observation windows, however (Fig. 2d), some cell cycles were incompletely or not recorded, hence some groups *g* were missing cells. In total, there were 252 exploitable distributions, corresponding to 4 cell types across 5 cell generations or less (depending on the dataset) and 4–6 cell features (depending on the cell cycle and the availability of *A*_*g*_ and *B*_*g*_) in each one of the 5 embryos (Fig. 3d–f and Supplementary Figs 3 and 4). Graphical assessment of these histograms, supported by a statistical test (Supplementary Figs 5 and 6), led to their classification into normal distributions for cell cycle lengths and mitosis times, and log-normal distributions for average volumes and surfaces. This allowed us to calculate the mean *μ* and standard deviation *σ* of each random variable in each cell group *g* and provide an idealised parametric representation of the whole cohort’s dataset in the form of 252 (*μ, σ*) pairs.

From these statistical measures, we concluded that random variables were largely independent from each other along one cell lineage: we detected no significant correlations among single features (temporal or spatial) of mother, daughter and sister cells (Supplementary Fig. 9 and Supplementary Table 3). Moreover, while the average cell cycle lengthened through generations with increasing fluctuations, the cell cycle length of a daughter did not depend on the cell cycle length of its mother. At the spatial level, we observed a variation in the range of ±20% around 0.5 for the average daughter/mother volume ratio, which mitigated the common assumption of average cell volume conservation across mitosis through consecutive generations at cleavage stages. However, although variability in volume and surface area at the individual cell level was not counterbalanced across consecutive generations, global uniformity could still be observed at the macroscopic level, as shown in Fig. 3 for the evolution of the total cellular volume.

### A multi-level probabilistic model relating individual cell features to embryonic dynamics

Based on this data analysis, we were able to formally relate individual cell features to embryo-wide features via a multi-level probabilistic model of the cell lineage (Fig. 1d). At its core, it consisted of positing the following three recursive relationships: *M*_*g*_ = *M*_*g*−1_ + *X*_*g*_, 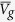 = 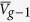 *A*_*g*_ and 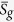 = 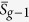 *B*_*g*_ where *g* − 1 = (*n* − 1, *k*) denotes the mother group of *g* (Supplementary Table 3–5). To evaluate this model, we calculated the differences between the empirical distributions (*M*_*g*_, 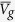, 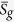) measured directly on the embryos’ final groups and their counterparts predicted from applying the iterative sum or product of (*X*_*g*_, *A*_*g*_, *B*_*g*_) on the first group. Among 48 eligible final groups, 75% of them showed a close proximity between measured and predicted parameter sets and 21% were in good agreement, confirming the accuracy of the model. In sum, we propose here a generic methodology to identify the probabilistic laws of the prototypic cell behaviours underlying blastula formation.

Next, using this model, realistic virtual cell lineages were generated and their statistics compared to the real data. Starting from the 32-cell stage, each simulated cell divided into two and its cell cycle length *x*_*i*_ was drawn from the idealised normal distributions (*μ*_*Xg*_, *σ*_*Xg*_). Given the relationships between cell features (Supplementary Table 5), similar to the relationships between random variables, the mitosis time of cell *i* was deduced by summing the cell cycle lengths of its ancestors *j*: *m*_*i*_ = ∑ *x*_*j*_. For each embryo, 300 realisations of the cell lineage were produced by varying the random generator’s seed. Comparison between these simulated specimens and empirical values on the three macroscopic quantities *N, W* and *Z* showed that embryo-level dynamics for each cell type was accurately reproduced using the empirical parameters measured in each cell group (Fig. 3g–i and Supplementary Fig. 11).

The number of cells over time *N*(*t*) displayed an alternation of plateaus, when no cells divided, and rapid increase during periods of high mitotic activity (Fig. 3a). During these periods, the slope of *N*(*t*) reflected the spread of mitoses over time, which our model showed to be caused by continuously desynchronising cell cycles along the lineage, and whose variance of division times within a cell group was equal to the sum of variances of cell cycle lengths in ancestor groups (Supplementary Note 2.3.1). In other words, we showed that the variability of division times came from accumulated variations in cell cycle lengths along the cell lineage. This result contrasts with previous statements about putative successive periods of synchrony, metachrony and asynchrony^21^. Moreover, there seemed to be no need for a “mitotic gradient” to explain the spatial distribution of mitoses^21^, as our study suggested that it could simply arise from the variability of cell cycle lengths.

The total cellular volume *W*(*t*) was globally conserved while undergoing alternating phases of contraction and expansion, which were also interpreted as emerging from the collective behaviour of individual cells characterised by desynchronising cycles but otherwise similar individual dynamics throughout these cycles (Fig. 3b and Supplementary Fig. 7). The total cellular surface area *Z*(*t*) followed a comparable evolution, but increased globally as cells became more cylindrical (Fig. 3c and Supplementary Fig. 8).

### Designing a data-driven prototype

In sum, each specimen of the cohort could be represented by a reduced number of parameters, the (*μ, σ*) pairs, sufficient to reproduce the embryo-level dynamics. To obtain a unified view of development during cleavage stages, a *prototypical representation* of the cohort was defined as the “centroid” of the five specimens in parameter space (Fig. 1g)^22^. This methodology was also used to define prototypical statistics for each cell group (black bars in Fig. 3d–f and Supplementary Figs 3 and 4), where intra-individual variability was represented by standard deviations computed for each cell feature. Similar to individual specimens, cell lineages were also simulated based on these statistics and the resulting prototypical embryo-level dynamics provided a representation of the “normal” development of sea urchin blastula (Figs 1h and 3j–l).

### Spatial embedding through biomechanical modelling

**Figure 4:**
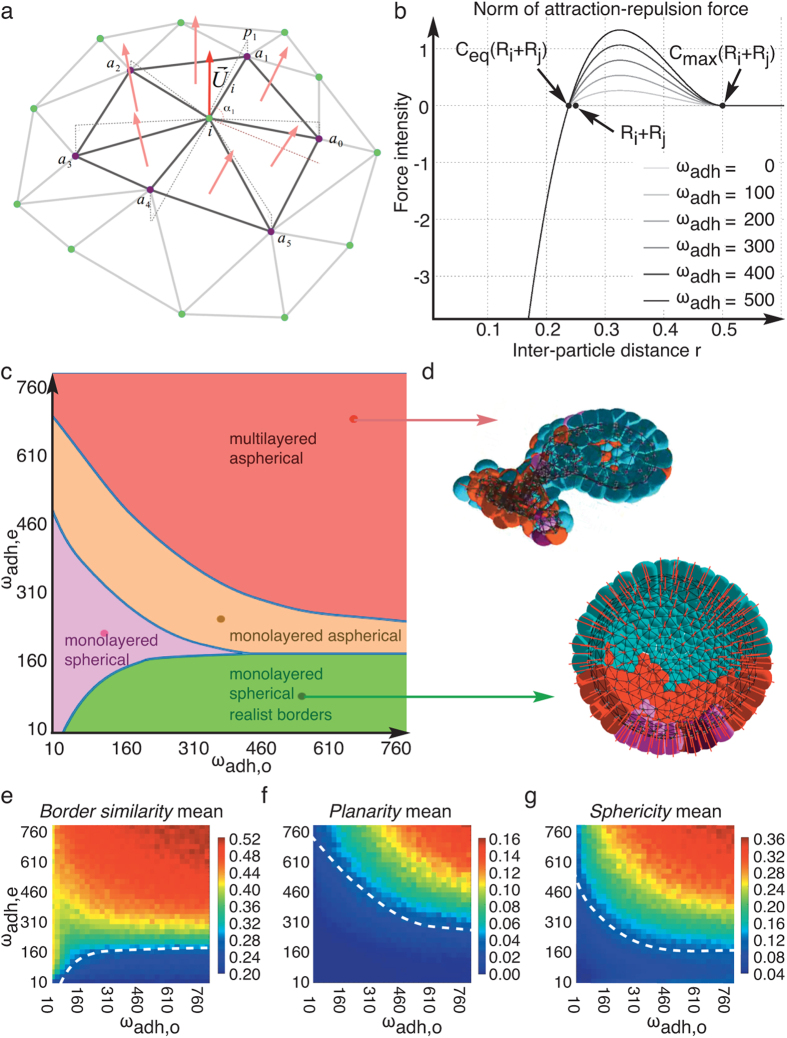
Spatial embedding in a biomechanical model of the prototypical multi-level probabilistic model. (**a**) Principle of cell axis computation. (**b**) Intensity of the attraction-repulsion force as a function of the interparticle distance *r*, with 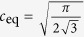 and 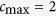. (**c**) Phenotypic phase diagram: the similarity of the simulation with digital embryos is computed for various pairs of homotypic and heterotypic adhesion coefficients (*ω*_adh,o_, *ω*_adh,e_). The green parametric region corresponds to the domain of best fit (Supplementary Video 2 shows simulations of representative embryos for each region). (**d**) Upper panel: example of multilayered aspherical “deviant” embryo. Lower panel: most realistic embryo. For both, section along the sagittal plane. (**e** to **g**) Landscapes of the three objective functions: cell type population border similarity *D*_*s*_, epithelial layer planarity *P*_*s*_, and embryo shape sphericity *S*_*s*_ (details concerning the calculation of these three metrics are described in the supplementary material). The fitness colour map goes from blue (best values) to red (worst values). The white dotted lines delimit the domains where the differences between observed and simulated embryonic developments are minimal. These lines are reproduced in the general phase diagram of (**c**).

In a last step, to understand the relations between individual cell features and the actual shape of the embryo, the cell lineage prototype was embedded in space via a biomechanical model (Figs 1i and 4). Here each cell was represented by a cylindrical particle oriented along the apicobasal axis of the epithelium (Fig. 4a and Supplementary Fig. 12). As cells are extremely small and sticky, inertia was negligible with respect to viscosity and cell displacements could be represented by an overdamped equation of motion (Supplementary Note 3.1.1). The epithelialisation of the forming blastula led to a decomposition of the forces exerted between neighbouring cells into tangential components and normal components, respectively: “attraction-repulsion” forces maintaining the integrity of the cell volume by stiffness and adhesion (Fig. 4b and Supplementary Fig. 13); and “planarity conservation” forces maintaining the monolayered structure of the epithelium and modelling the blastocoel turgor pressure (Supplementary Fig. 14). In the first case, the adhesion coefficient *ω*_adh_ could take two values, homotypic (*ω*_adh,o_) or heterotypic (*ω*_adh,e_), depending on the match between the two cell types in contact. In addition, cell division was oriented inside the tangential domain at an angle drawn randomly from a uniform distribution.

### Fitting the free parameters to the experimental data

A parameter space exploration in the (*ω*_adh,o_, *ω*_adh,e_) plane (Fig. 1j–l) revealed a “phase portrait” characterised by multiple phase transitions between different phenotypic domains (Fig. 4c,d): from highly spherical and planar embryos to collapsed cell aggregates, via polylobular structures appearing in a region of high planarity without sphericity (Supplementary Table 6 and Supplementary Video 2). The validation or rejection of these virtual specimens relied on an “objective function”, or fitness, describing the layout of the embryos and comparing it to the ones reconstructed from the *in vivo* data (Fig. 4e–g). It was composed of three terms: a degree of sphericity of the global shape, a degree of planarity of the monolayered epithelium, and a degree of similarity of the borders between cell populations (Mic/Mac and Mac/Mes) with the real specimens. The domain of best fit, i.e. the one that governed the most realistic spatial model of the sea urchin embryo development, was obtained for a low heterotypic adhesion coefficient *ω*_adh,o_, confirming that clear-cut borders could emerge without the need to bias the division orientation. Moreover, when the attraction/planarity force ratio was too high, the embryo collapsed and the monolayered epithelium folded into a 3D aggregate (Fig. 4d and Supplementary Video 2).

## Discussion

We have shown that the characteristics of cell proliferation and cell interactions, as quantified and modelled here, provided a structurally constrained process that guaranteed the robust formation of the blastula. Altogether, modelling, simulation and parameter space exploration brought insights into the developmental constraints underlying morphogenetic processes, such as cell adhesion and blastocoel turgor pressure, otherwise difficult to quantify *in vivo*. Postulating differential homotypic and heterotypic adhesion forces without modulating the parameters as a function of cell types was suitable to reproduce the geometry and organisation of the blastula. This is consistent with dissociation and reaggregation experiments at the blastula stage that do not formally imply cell sorting of the different cell populations of the blastula^23^. Moreover, in the hypothesis of similar adhesion forces within the different cell populations, one would expect different E-cadherin densities to compensate for specific cell sizes and surface contacts^24^,^25^. This could effectively lead to a significant difference between homotypic and heterotypic adhesion parameters, as exhibited by our model.

The invariance of the measured and modelled cell behavioural rules during the observation window suggests that the embryo of *P. lividus* is under a single dynamical regime where cell cycle asynchrony continuously increases and volumes converge to uniform values. The highlighted interplay of homogenisation and diversification at cell population and individual cell levels does not, however, presume that a precise regulation of tissue patterning is already operating at blastula stages at the subcellular level. The current view of a finely tuned regulation of gene network dynamics during sea urchin early embryogenesis may hide similar individual cell variability and population-level averaging effects^26^. The extent of variability of gene expression, its importance in the dynamics of the molecular and genetic interaction networks, and its consequences at the level of cellular dynamics remain open and challenging questions^2^,^27^.

The generic methodology presented here contributes to setting the foundations for developmental biology as a quantitative science, integrating multi-level data and allowing the formulation of theoretical hypotheses to be tested experimentally. Using this framework, we can address biological questions by considering perturbative experiments. Parameters of the model, such as cell stiffness, cell adhesion and blastocoel turgor pressure, can be modified experimentally and results compared to the simulations to test the validity of the predictions. The statistical approach elaborated in this paper to characterise the cell lineage of various individual organisms is general enough to be applied to other species. The cell branching process that gives rise to the cell lineage is observed in many different organisms. Our mathematical formulation is directly applicable to organisms undergoing periods of cleavage during their development, where cells’ main activity is to divide. Our MATLAB library is readily available to analyse digital cell lineage trees and produce the corresponding prototypical stochastic cell lineages annotated with cell volume and cell surface measurements.

## Methods

### Embryo staining, mounting and imaging

Oocytes from *Paracentrotus lividus* were prepared and injected^28^ with 150 μg/ml H2B-mCherry and 150 μg/ml eGFP-HRAS synthetic mRNA. Embryos were maintained between slide and coverslip covered with protamine. Imaging was achieved with a Leica upright microscope (DM5000) SP5 MLSM equipped with a 20x/0.95NA W or 63x/0.9NA dipping lens objective. Details of image acquisition are provided in Supplementary Table 1. Simultaneous 2-photon excitation^29^ at two different wavelengths (1030 nm and 980 nm) was performed by pulsed laser beams (T-pulse 20 by Amplitude Systèmes, and Ti-Sapphire femtosecond oscillator MaiTai HP by Newport Spectra physics, respectively).

### Digital embryo reconstruction

Reconstructed embryos were obtained by running the raw image datasets through the automated image processing workflow of the BioEmergences platform. A detailed description of the reconstruction methods, including the mathematical algorithms used, was published previously^6^ (Supplementary Note 1). The three main components described in the next sections are: nucleus centre detection, cell trajectory tracking, and cell membrane segmentation.

### Nucleus centre detection and tracking

Cell positions were placed at the local intensity maxima of cell centre images filtered by a difference of Gaussians (Supplementary Note 1.1). Tracking of the cell nuclei was obtained by first constructing tree branches connecting nearest cells on consecutive images when that proximity criterion was reciprocal. Then, mitoses were detected by linking the cells left over without a predecessor to their closest neighbour at the previous time step. Finally, the resulting lineage tree was refined via the minimisation of a functional defined by an energy term based on the assumption that the embryo essentially behaves like an elastic mass (i.e. that neighbour cells at time *t* remain close at time *t* + 1). This functional was minimised using a simulated annealing heuristic, searching for the lowest-energy configuration by randomly changing a link, removing/adding a link or removing/adding a cell. To validate the tracking, all cell positions and trajectories were checked by at least two experts through visual inspection, comparing the digital reconstruction with the raw data using our Mov-IT visualisation software^6^. All detectable errors (false positive and false negative nuclei, false links, missing links, false positive and false negative mitosis events) were corrected to build error-free lineages. All five lineage trees were fully validated (Supplementary Note 1.2 and 1.3).

### Cell shape segmentation

To remove the noise and smooth the images while faithfully preserving edge information, the data was first filtered by geodesic mean curvature flow (GMCF)^30^. Cell shapes were then obtained by applying a generalised version of the subjective surface (SubSurf) method^31^,^32^ for its ability to reconstruct missing boundaries, making it particularly suitable for this type of data (Supplementary Note 1.4).

### Temporal and spatial rescaling

The measured number of cells, total cellular volume and total cellular surface area presented similar patterns of evolution without overlapping. To filter out this variability and proceed to further inter-individual comparison, we applied temporal and spatial rescaling functions. The time dependency of each embryo was rescaled using an affine transformation, whose parameters were obtained by minimising the difference between the cell-number curve and the averaged number of cells over the cohort. Spatial rescaling is a linear transform performed along each spatial dimension. For each embryo, a custom tuning parameter was obtained by minimising the difference between the temporally rescaled cellular volume of an embryo and the mean value over the cohort (Supplementary Note 2.1.3).

### Individual cell features

Individual cell features were extracted from the reconstructed and rescaled cell lineages and segmentations. For a cell *i*, we defined: its cell cycle length *x*_*i*_, its division time *m*_*i*_, its volume *v*_*i*_(*t*) and surface area *s*_*i*_(*t*) at each time step, with their averaged values 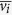 and 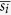 between two consecutive mitoses, the ratio 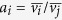 between its average volume and that of its mother *j*, the ratio 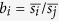 between its average surface area and its mother’s, the number *d*_*i*_(*t*) of neighbours (or “degree”) in its network of cell contacts, the number *n*_*i*_ of cell cycles (or “generation index”) that it has undergone since fertilisation including the current one, and its cell type 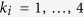 corresponding to Mes, Mac, LMic, SMic, respectively (Supplementary Note 2.1.1).

### Cell feature distributions in cell groups

Symmetries in the embryo, such as the rotational symmetry around the animal-vegetal (AV) axis, prevent the identification and matching of individual cells from one specimen to another. Unique identification of cells based on their morphological characteristics cannot be done without ambiguity either. To overcome these obstacles, cell features were binned into mesoscopic groups defined by common generation *n* and type *k*, denoted by *g* = (*n, k*). For example, we considered the distribution *X*_*g*_ of cell cycle lengths for 32 Mes cells at the 7^th^ generation, i.e. for *g* = (7, 1). From these empirical distributions of individual cell features in each cell group, we derived idealised normal or log-normal representations parameterised by a mean-variance pair (*μ, σ*), for example: 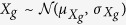. The accuracy of modelling distributions of cell features with normal distributions (for cell cycle lengths {*x*_*i*_} and division times {*m*_*i*_}) or log-normal distributions (for cell volumes 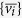, surface areas 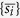 and their associated daughter/mother ratios {*a*_*i*_} and {*b*_*i*_}) was assessed using a chi-square goodness-of-fit test. Among 252 available distributions, 124 were eligible for statistical analysis due to the relatively small sample size. Only 20 of them had a p-value under 0.05, among which 12 were under 0.01. The complete ranges of p-values obtained were the following: [3.7e-8, 0.42] for cell cycle lengths {*x*_*i*_}, [3.6e-6, 0.46] for mitosis times {*m*_*i*_}, [8.5e-3, 0.89] for average cell volumes 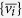, [5e-3, 0.96] for average cell surface areas 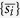, [0.06, 0.84] for average volume ratios {*a*_*i*_}, and [1.3e-3, 0.76] for average surface area ratios {*b*_*i*_}. More details are provided in Supplementary Note 2.2.1.

### Independence within the lineage

To investigate the degree of correlation between cells belonging to the same descent, we tested linear dependencies between cell distributions of daughters and mothers, and among sisters for temporal features (division times and cell cycle lengths) and spatial features (volumes and surface areas). We computed Pearson’s correlation coefficient *R*^2^ between distributions. Given the weak values of *R*^2^ obtained in the different cell groups (*R*^2^ was greater than 0.6 in only 20 cases out of 198 investigated pairs of distributions), we adopted the viewpoint that the various random variables describing cell features’ distributions were independent between and within cell groups (Supplementary Note 2.2.3).

### Multi-level probabilistic model

To design a probabilistic model, we used the parameterised description of individual feature distributions obtained in each cell group together with the assumption of independence among the different groups. The equations describing the temporal relations between cell features in the lineage tree are:


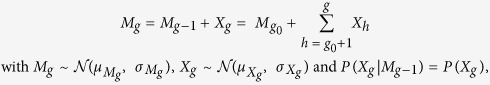


where *M*_*g*_ and *X*_*g*_ are the random variables describing the probability distributions of division times and cell cycle lengths, respectively; (*μ, σ*) are the mean and standard deviation of a normal distribution; and *g* ± 1 is a shortcut notation for (*n* ± 1, *k*). The last equation indicates the independence of the cell cycle from the division time of its mother. Similarly, the equations describing the spatial relations between cell features in the lineage tree are:


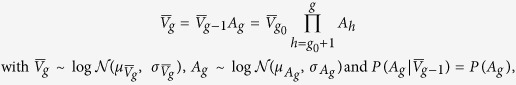


where 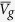 and *A*_*g*_ are the random variables describing the probability distributions of average cell volumes and daughter/mother volume ratios, respectively; and (*μ, σ*) are the mean and standard deviation of a log-normal distribution. The last equation indicates the independence of the cell daughter/mother volume ratio from the volume of its mother. We obtained similar equations for the surface area. The detailed derivation of the multi-level probabilistic model is provided in Supplementary Note 2.3.

### Model evaluation

To evaluate the accuracy of this multi-level probabilistic model, we computed the predicted parameters values (*μ, σ*) for the probability distributions of division times *X*_*g*_, average volumes 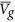 and surface areas 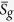 in the last cell cycle of each embryo using recursive equations derived from the ones above (Supplementary Note 2.3.1 and 2.3.2). These estimated parameters were compared to the actual ones using the Kullback-Leibler divergence *D*_*KL*_. The value of the comparison was symmetrised and normalised by averaging the differences between the embryos of the cohort (using the same measure of dissimilarity), and denoted by 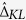. Among the 48 eligible groups, 75% showed a close proximity (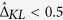 in 36 cases), 21% were in good agreement (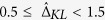 in 10 cases), and 4% fell far away (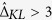 in two cases: for 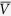 and 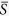 in the Mes cells of embryo #5). Therefore, except for these two outliers, our probabilistic model accurately predicted the dynamics of sea urchin development (Supplementary Note 2.3.4).

### Prototype

The multi-level probabilistic model provided an invariant framework relating individual cell features to embryo-level dynamics with specific parameter values for each embryo. To obtain a unified representation, or “prototype”, of the sea urchin blastula’s normal development, individual measurements were aggregated and averaged over the cohort into a “centroid” (a virtual average embryo) at the level of cell groups. This centroid was computed using the normalised symmetrised Kullback-Leibler divergence 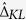 for each individual cell feature distribution (Supplementary Note 2.4).

### Simulation of artificial cell lineages

Using the multi-level probabilistic model, realistic virtual cell lineages were generated and their statistics compared to the real data. Starting from the 32-cell stage (comprising 16 Mes, 8 Mac, 4 LMic, and 4 SMic cells), a cell lineage was computed by letting each cell divide into two and implementing the relationships between microscopic features derived from the relations between random variables (Supplementary Table 5), then using the estimated values of the parameters for each cell group, in every embryo and in the prototype (Supplementary Notes 2.3.5 and 2.4).

### Spatial modelling

Relying on the above digital reconstruction and probabilistic model, the last stage of our study consisted of a spatially explicit computational model and simulation of the sea urchin embryo based on the MecaGen platform^18^. In a nutshell, it was designed as a particle-based model, where the interactions between cells were decomposed into an attraction-repulsion component and a planarity conservation component. The attraction-repulsion force 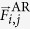 exerted by cell *j* on cell was defined as follows:


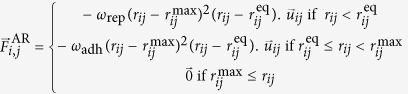


where *r*_*ij*_ is the distance between *i* and *j*; 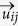 is the neighbourhood vector from *j* to *i*; 
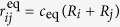
 with 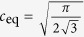 is an equilibrium distance depending on the cells’ radii *R*_*i*_ and *R*_*j*_; 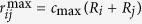 with 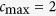 is the maximum distance of contact between two cells; and *ω*_rep_ and *ω*_adh_ are adhesion coefficients tuning the repulsive and adhesive parts of the force profile. The planarity conservation component 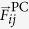 was defined as: 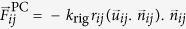, with 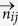 being the bisector of each cell’s normal vector (vector pointing from the centre of the embryo towards the outside) and *k*_rig_ is a planar rigidity coefficient. More details on the derivation and implementation of the model can be found in Supplementary Note 3.1.

### Estimation of free parameters

In the model several parameters have values that cannot be directly measured. The values of the free parameters leading to the most realistic spatial modelling and embedding of the prototypical cell lineage can be obtained by finding the best fit between the spatially explicit simulations and the original data. To this end, we designed a metric based on the shape of the boundaries between cell populations (obtained through the network of cell contacts) and two “objective functions” characterising certain properties of the simulated embryos: their ability to remain monolayered during development and their sphericity. The values of the repulsion coefficient *ω*_rep_ and the planar rigidity coefficient *k*_rig_ were fixed. The attraction-repulsion force adopted different values depending on whether the cells belonged to the same population (via a homotypic adhesion coefficient *ω*_adh,o_) or to different populations (via a heterotypic adhesion coefficient *ω*_adh,e_). Various values of *ω*_adh,o_ and *ω*_adh,e_ were systematically explored in a 2D domain of parameter space (from 10 to 1000 with an increment of 25 for each parameter), by simulating 300 embryos in each point and comparing them to the original data via the above metric (Fig. 4c and Supplementary Note 3.2.3).

### Software and data availability

The BioEmergences workflow was published as a standalone software^6^. The MecaGen modelling platform was published as an open source software^18^. The scripts for the statistical analysis and for the construction of the prototype from empirical data are provided here as a MATLAB library (Supplementary Software 1). The compressed folder contains a preprocessed version of the five sea urchin datasets. The raw data is made publicly available on the BioEmergences website:

http://bioemergences.eu/bioemergences/pubs/seaurchin

## Additional Information

**How to cite this article**: Villoutreix, P. *et al*. An integrated modelling framework from cells to organism based on a cohort of digital embryos. *Sci. Rep.* **6**, 37438; doi: 10.1038/srep37438 (2016).

**Publisher's note:** Springer Nature remains neutral with regard to jurisdictional claims in published maps and institutional affiliations.

## Supplementary Material

Supplementary Information

Supplementary Video 1

Supplementary Video 2
