# Supplementary material for: An integrated modelling framework from cells to organism based on a cohort of digital embryos
Source: Sci Rep. 2016 Dec 2;6:37438. doi: 10.1038/srep37438 (PMC5133568; doi:10.1038/srep37438)
Supplement: Supplementary Information [file srep37438-s1.pdf]

Supplementary Material

An integrated modelling framework from cells to organism based on a cohort of digital embryos

Paul Villoutreix[1,2,3,*], Julien Delile[1,2,4,*], Barbara Rizzi[1,2,5,*], Louise Duloquin[1,2,*], Thierry Savy[1,2], Paul Bourgine[1,2], René Doursat[1,2,6], and Nadine Peyriéras[1,2,†]

[1]BioEmergences USR3695, CNRS, Université Paris-Saclay,
Avenue de la Terrasse, 91198 Gif-sur-Yvette Cedex, France.
[2]Institut des Systèmes Complexes Paris Ile-de-France (ISC-PIF) UPS3611, CNRS,
113 rue Nationale, 75013 Paris, France.
[3]Present: Lewis-Sigler Institute for Integrative Genomics, Princeton University, Princeton, NJ 08544, US
[4]Present: The Francis Crick Institute, Mill Hill Laboratory, Mill Hill, London NW7 1AA, UK.
[5]Present: TEFOR Core Facility, Paris-Saclay, Institute of Neuroscience UMR9197, CNRS,
Université Paris-Sud, 91190 Gif-sur-Yvette, France.
[6]Present: Informatics Research Centre (IRC), School of Computing, Mathematics & Digital Technology,
Manchester Metropolitan University, John Dalton Building, Chester Street, Manchester M1 5GD, UK.

[*]These authors contributed equally to this work.
[†]To whom correspondence should be addressed. E-mail: nadine.peyrieras@cnrs.fr

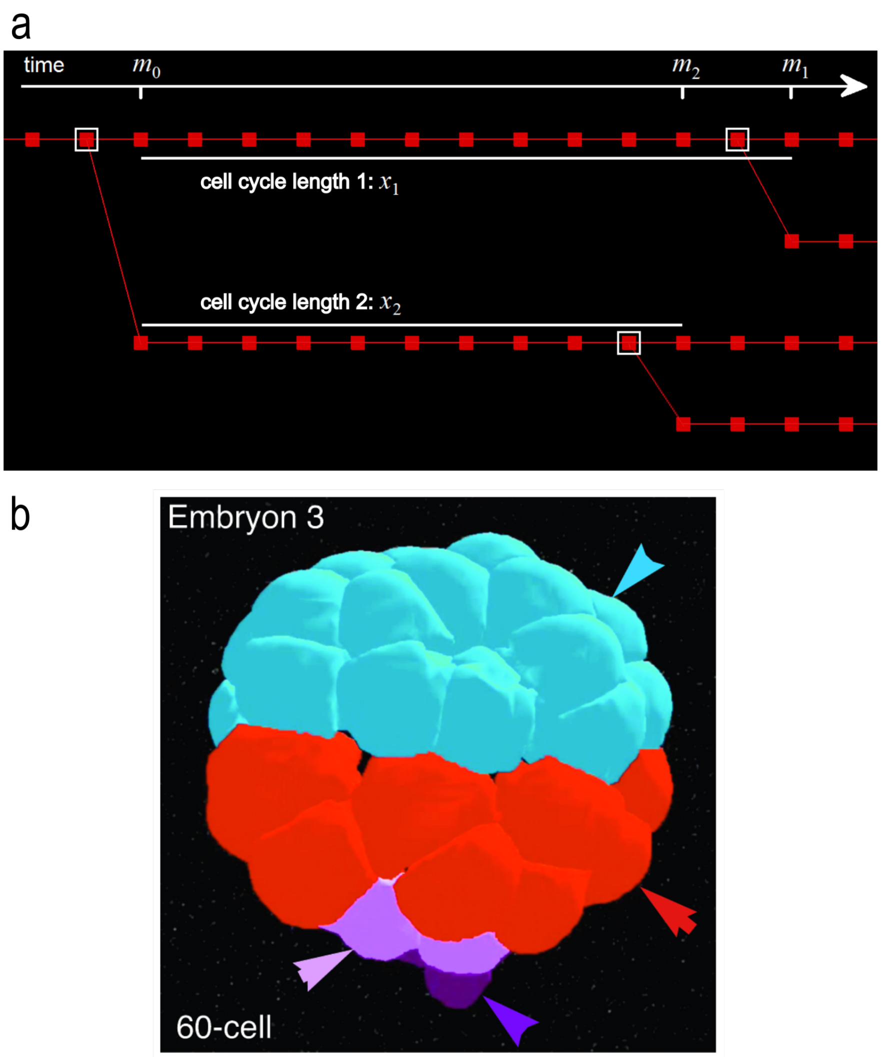

Supplementary Figure 1: (a) Sample of cell lineage indicating how cell cycle lengths are calculated. Each node (red square) represents the state of one cell at an instant t. A highlighted node (white square) corresponds to the last state of a cell just before the detection of mitosis. The subset of bifurcation nodes form a regular binary tree, while the number of intermediate nodes on one branch is variable and depends on the distribution of cell cycle lengths. (b) The four cell populations identifiable at the 32-cell stage, from top to bottom: 16 mesomeres (Light Blue), 8 macromeres (Red), 4 large micromeres (Magenta), and 4 small micromeres (Violet).

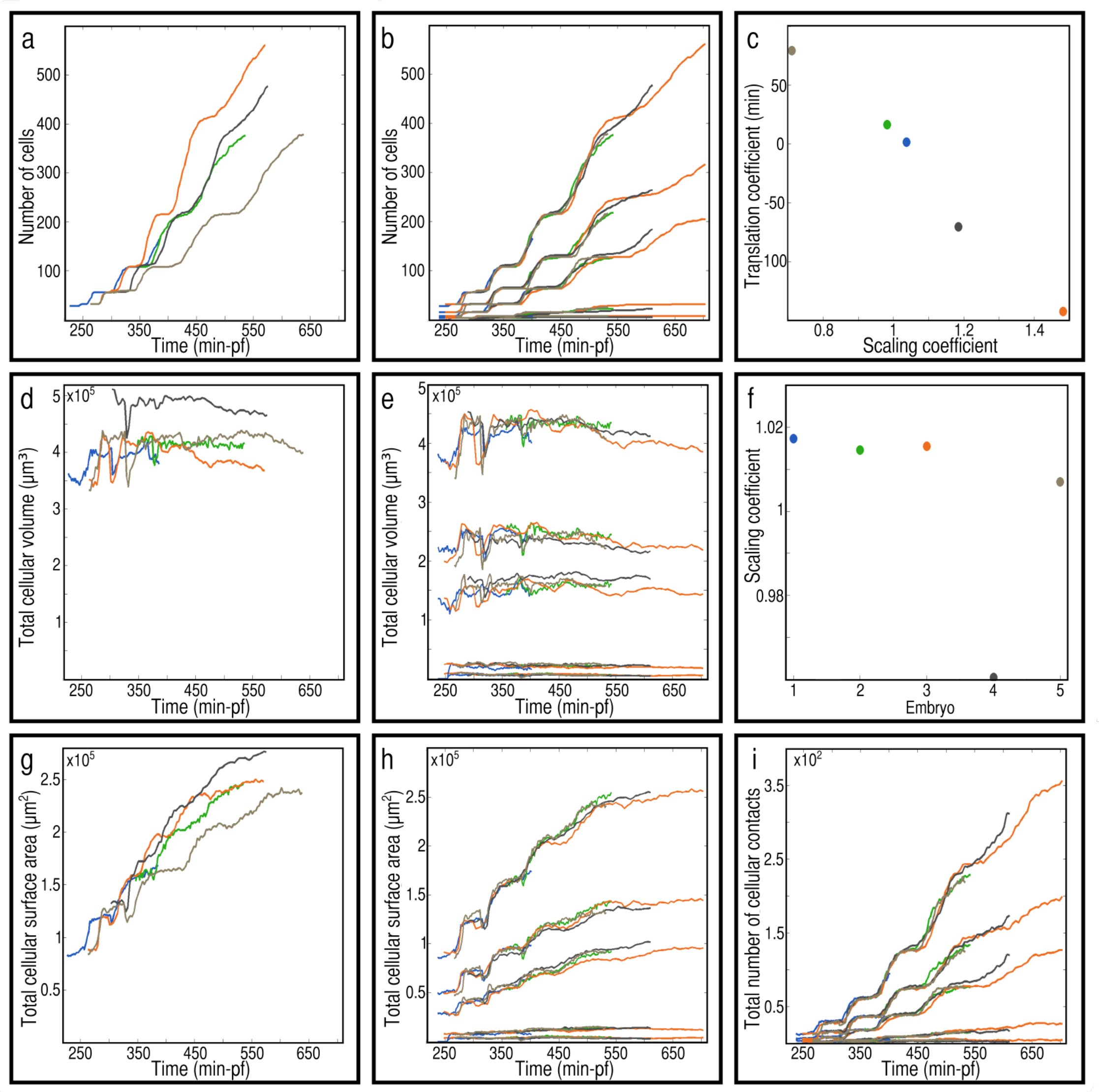

Supplementary Figure 2: Embryo-level dynamics before and after temporal and spatial rescaling. Starting from the 32-cell stage, the global measures of five specimens $e = 1,\ldots,5$ are plotted over their respective imaging periods. a) Number of cells $N_e(t)$ in each embryo. Original periods in minutes post-fertilization (mpf): 225-396 mpf (blue), 345-507 mpf (green), 260-588 mpf (orange), 300-575 mpf (cyan), and 260-638 mpf (pink). b) Temporally rescaled number of cells in each embryo, $N_e(\alpha_e t + \beta_e)$ (top curves), and each cell population, $N_e^k(\alpha_e t + \beta_e)$ (four groups of lower curves, from top to bottom: Mes, Mac, LMic, SMic). The rescaled periods are: 240-398 mpf (blue), 357-539 mpf (green), 246-702 mpf (orange), 291-619 mpf (cyan), and 265-531 mpf (pink). c) Affine transform parameters (α_e, β_e) used in the previous top curves. d) Total cellular volumes $W_e(t)$. e) Temporally and spatially rescaled total cellular volumes, $(\gamma_e)^3 W_e(\alpha_e t + \beta_e)$ (top), and field cellular volumes $(\gamma_e)^3 W_e^k(\alpha_e t + \beta_e)$ (same order as b), using the same parameters (α_e, β_e) as before. f) Coefficients γ_e used in the previous top curves. g) Total cellular surface areas $Z_e(t)$. h) Temporally and spatially rescaled total cellular surface areas, $(\gamma_e)^2 Z_e(\alpha_e t + \beta_e)$ (top), and field cellular surface areas $(\gamma_e)^2 Z_e^k(\alpha_e t + \beta_e)$ (same order as b), using the same parameters (α_e, β_e) and γ_e as before. i) Temporally rescaled total numbers of contacts $C_e(\alpha_e t + \beta_e)$ and field numbers of contacts $C_e^k(\alpha_e t + \beta_e)$.

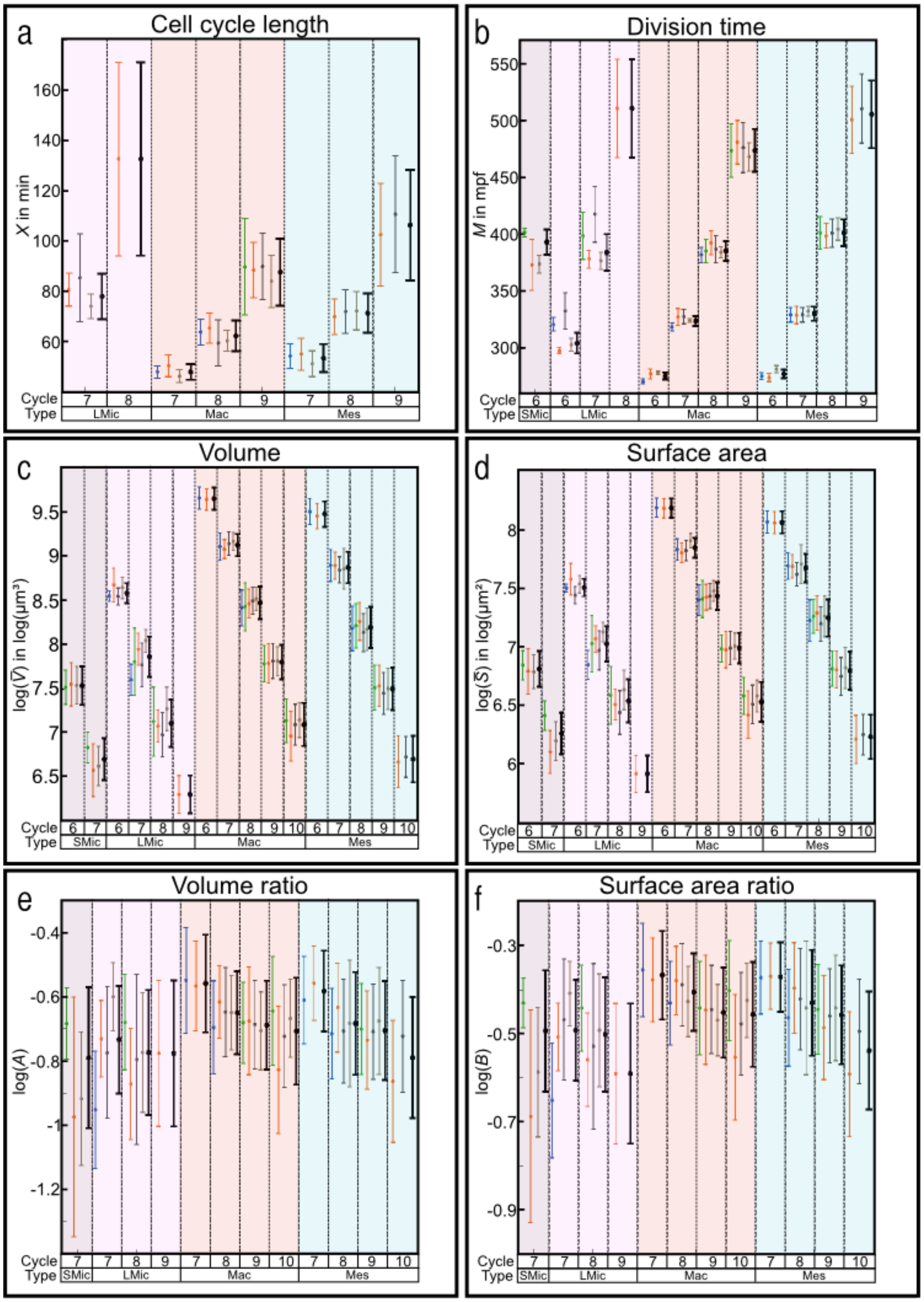

Supplementary Figure 3: Mean and standard deviation bars representing the normal and log-normal approximations of the cell feature distributions in various cell groups $\mathscr{L}_g$. For each feature (one per frame), the cell groups $g = (e, n, k)$ cover all four cell types k (except cell cycle length), one or several generations $n \in [6, 10]$ per type, and most of the five embryos e per generation-type combination (using the same colours blue, green, orange, cyan, pink for each embryo as Supplementary Fig. 2 with additional bars (black, bold) for the prototype).

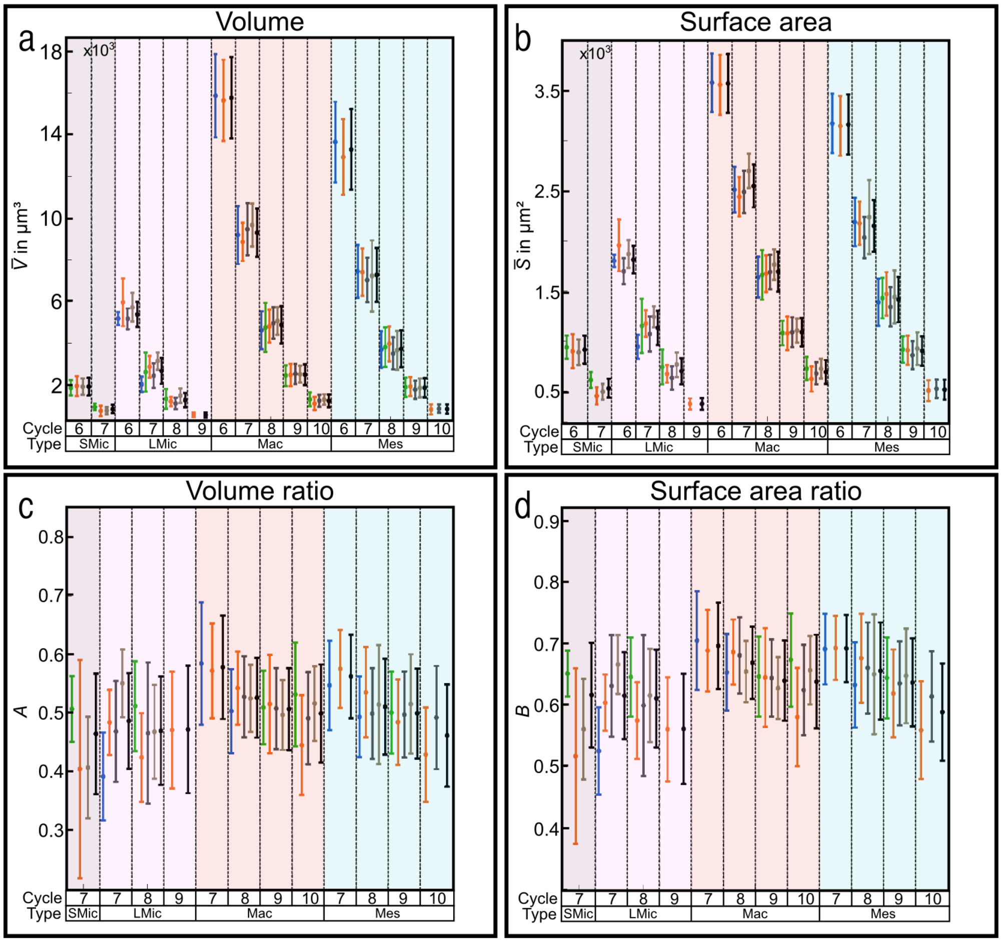

Supplementary Figure 4: Same as Supplementary Fig. 3 c to f without applying the log function to the volume and surface area. This figure is intended to highlight the value 0.5 in the daughter/mother volume ratio (here in c).

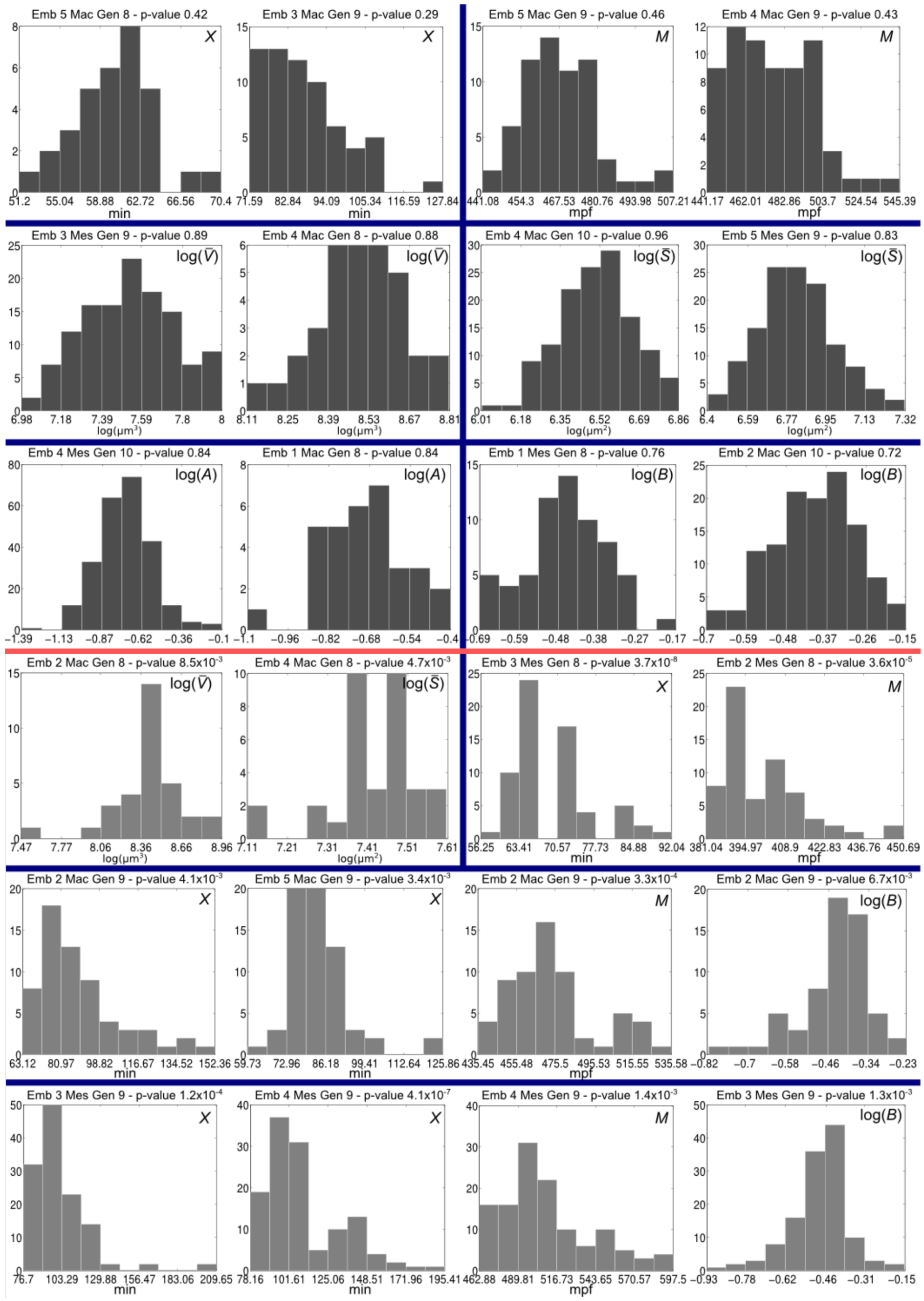

Supplementary Figure 5: **Top Panel** (above the red line) - The 12 cell feature distributions (two per variable type) with the highest p-value, i.e. the best cases of fit by normal or log-normal curves with respect to the chi-squared test. Left to right, top to bottom: the two X_g (cell cycle length) and two M_g (division time) distributions closest to normal curves; the two $\overline{V}_g$ (average volume), two $\overline{S}_g$ (average surface area), two A_g (daughter/mother volume ratio) and two B_g (daughter/mother surface area ratio) distributions closest to log-normal curves. Titles indicate the embryo e, cell type k, generation n and p-value. **Bottom Panel** (below the red line) - The 12 worst-fit cases (p-value < 0.01) of cell feature distributions, where the assumption of normal or log-normal curves could not be retained with respect to the chi-squared goodness-of-fit test. They fall into four n, k categories: 8, Mac; 8, Mes; 9, Mac; and 9, Mes. Most of them concern X (cell cycle length) and M (division time). - See Supplementary Note 2.2.1

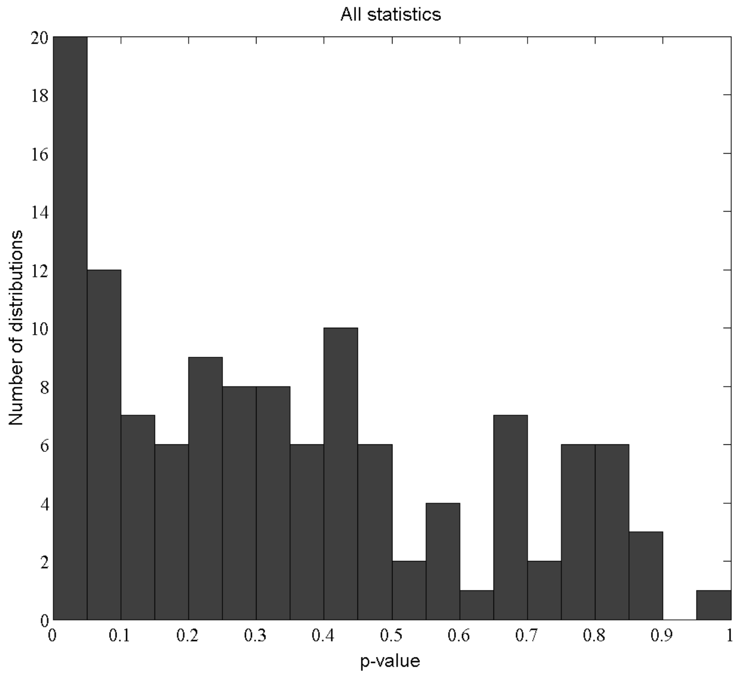

Supplementary Figure 6: Histogram of all p-values obtained by chi-squared goodness-of-fit test on 124 distributions of cell features. The six variables considered here were X_g (cell cycle length), M_g (division time), $\log \overline{V}_g$ (log average volume), $\log A_g$ (log average surface area), $\log \overline{S}_g$ (log daughter/mother volume ratio) and $\log B_g$ (log daughter/mother surface area ratio) across the five embryos e, four cell types k, and all generations n within the period during which the cell feature was observable. - See Supplementary Note 2.2.1

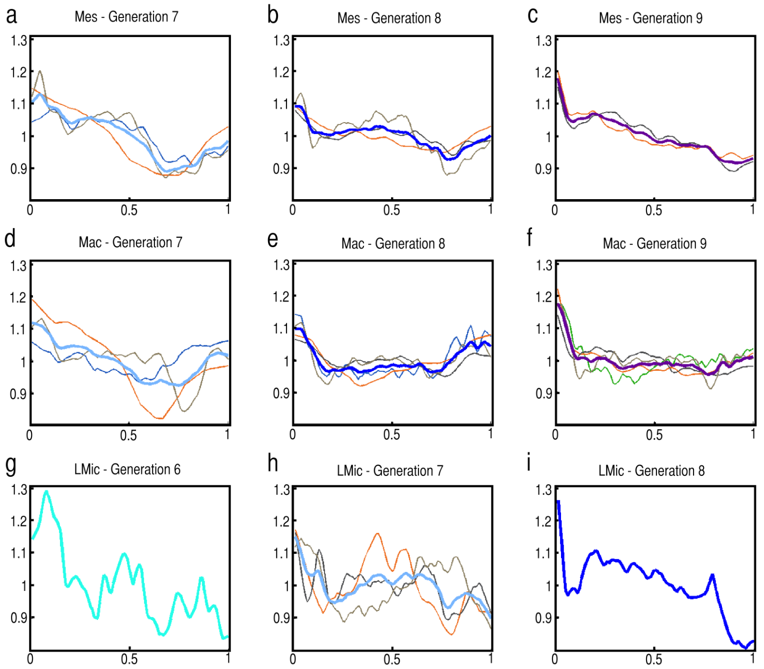

Supplementary Figure 7: Estimated cell volume microdynamics $\widetilde{\omega}_g(u)$ in various groups $\mathscr{L}_g$ of types $k = 1,2,3$ and generations $n = 6,7,8,9$. The volume of each individual cell has been normalized by its averaged volume and is considered as a function of percentage of elapsed cell cycle. The various individual cell volume dynamics have been averaged in each cell groups. Thin lines correspond to individual embryos e of the cohort $\mathscr{E}$ (one or several per group). Thick lines represent cohort averages $\langle \widetilde{\omega}_g(u) \rangle_{e \in \mathscr{E}}$. The rows from top to bottom correspond to the various cell types, Mesomeres, Macromeres, Large Micromeres and the columns correspond to the cell generations (7 to 9 for Mes and Mac and 6 to 8 for LMic). More details can be found in Supplementary Note 2.2.2.

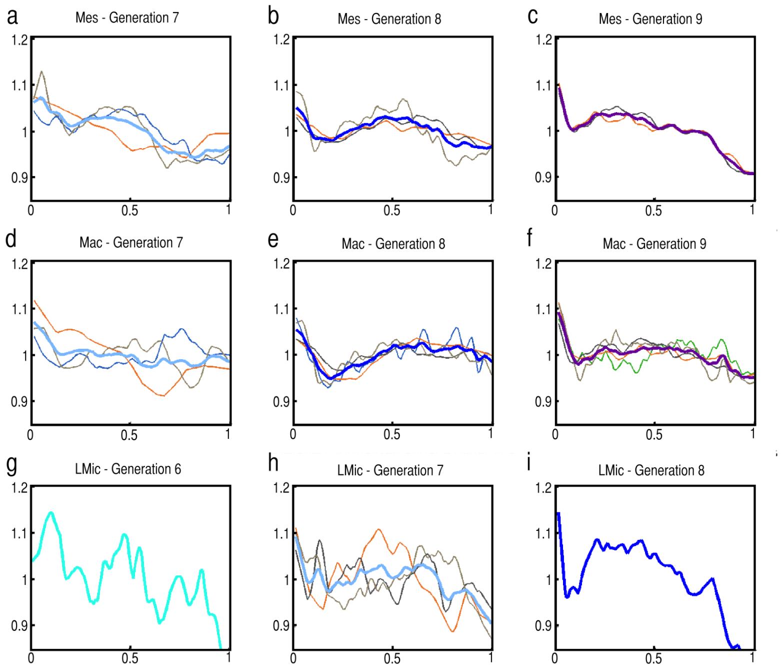

Supplementary Figure 8: Estimated cell surface area microdynamics $\widetilde{\phi}_g(u)$ in various groups $\mathscr{L}_g$ of types $k = 1,2,3$ and generations $n = 6,7,8,9$. The surface area of each individual cell has been normalized by its averaged surface area and is considered as a function of percentage of elapsed cell cycle. The various individual cell surface area dynamics have been averaged in each cell groups. Thin lines correspond to individual embryos e of the cohort $\mathscr{E}$ (one or several per group). Thick lines represent cohort averages $\langle\widetilde{\phi}_g(u)\rangle_{e\in\mathscr{E}}$. As on the Supplementary Fig. 7, the rows from top to bottom correspond to the various cell types, Mesomeres, Macromeres, Large Micromeres and the columns correspond to the cell generations (7 to 9 for Mes and Mac and 6 to 8 for LMic). More details can be found in Supplementary Note 2.2.2.

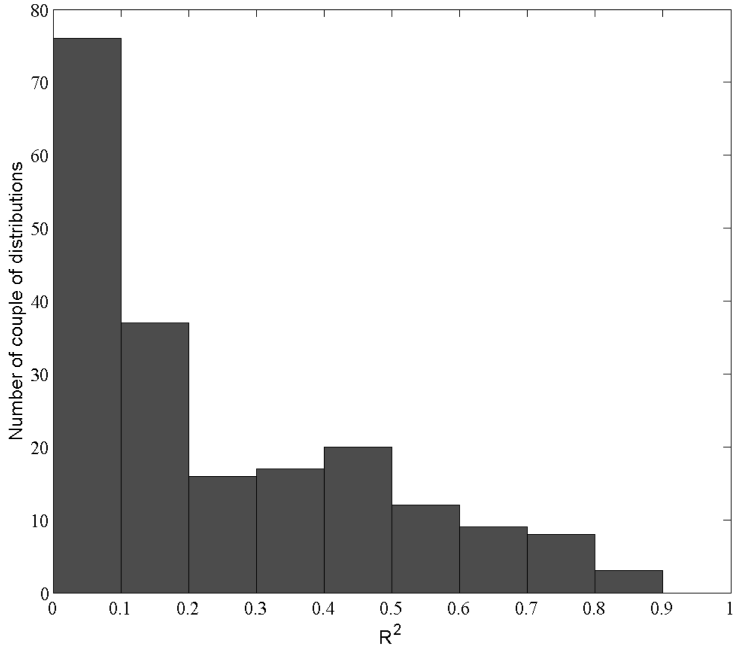

Supplementary Figure 9: Histogram of the coefficient of determination $R^2 = \widetilde{\rho}^2_{X,Y}$ for the various pairs of distributions X and Y displayed in Supplementary Table 3 and described in details in Supplementary Note 2.2.3. Only 20 values rank above 0.6 among 198 observed pairs of distributions.

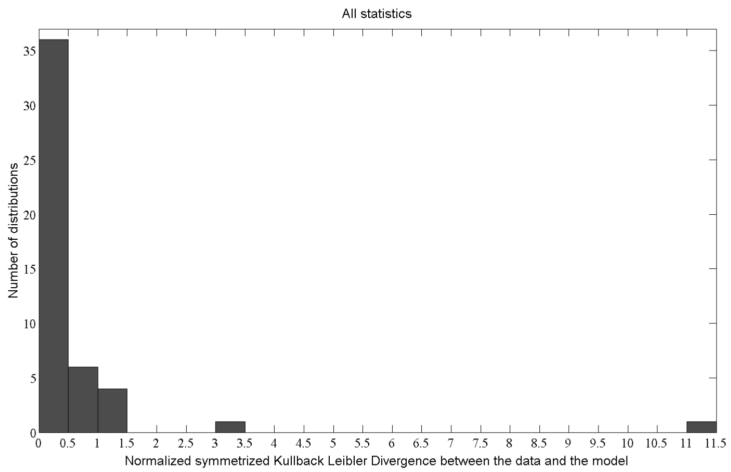

Supplementary Figure 10: Histogram showing the results of the evaluation of the proximity of the model to data using the normalized distance function $\widehat{\Delta}_{\mathrm{KL}}$ defined in equation 38. This evaluation is a comparison of 48 distributions predicted by the multi-level probabilistic model and measured empirically. More details can be found in Supplementary Note 2.3.4

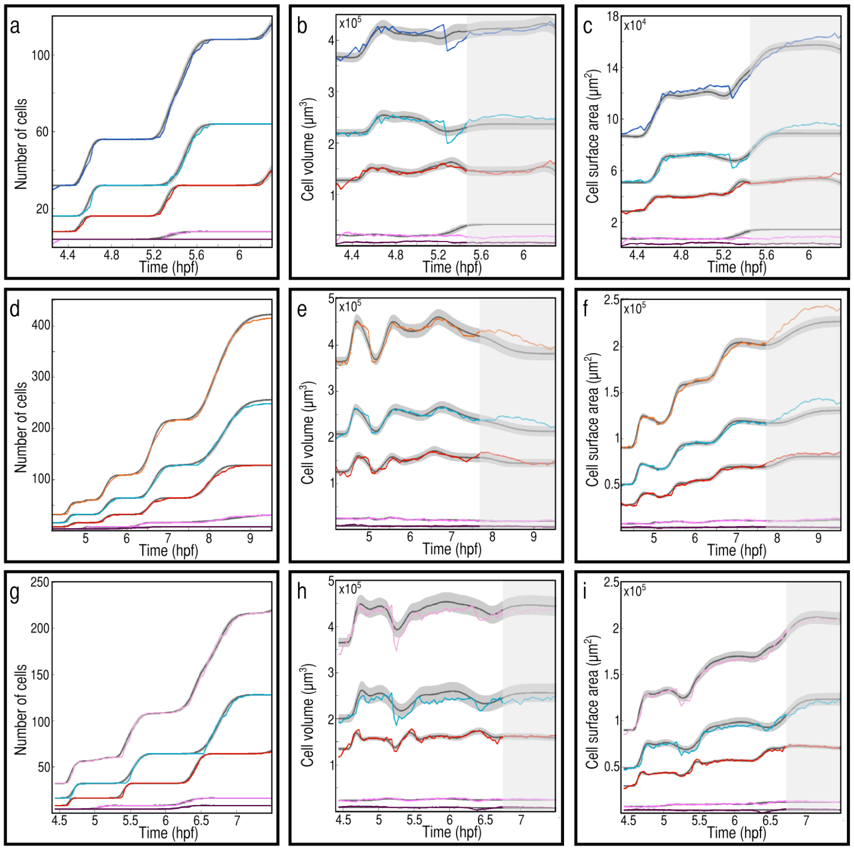

Supplementary Figure 11: Simulation of artificial cell lineages and comparison with real data. The three rows correspond to embryos 1, 3, and 5. The three columns represent the total number of cells $N(t)$, the total cellular volume $W(t)$ and surface area $Z(t)$. Inside each chart, the top curves show the embryo-level quantities (N_e, W_e, or Z_e), while the four groups of lower curves show their type-specific components (N_e^k, W_e^k, or Z_e^k), from top to bottom: Mes, Mac, LMic, SMic. In black: mean of 300 simulated cell lineages; in gray: their standard deviation; in colour: measured values in the real specimen (all curves obtained by temporal and spatial rescaling). Shaded areas: incomplete cycles, for which an accurate estimation was not possible.

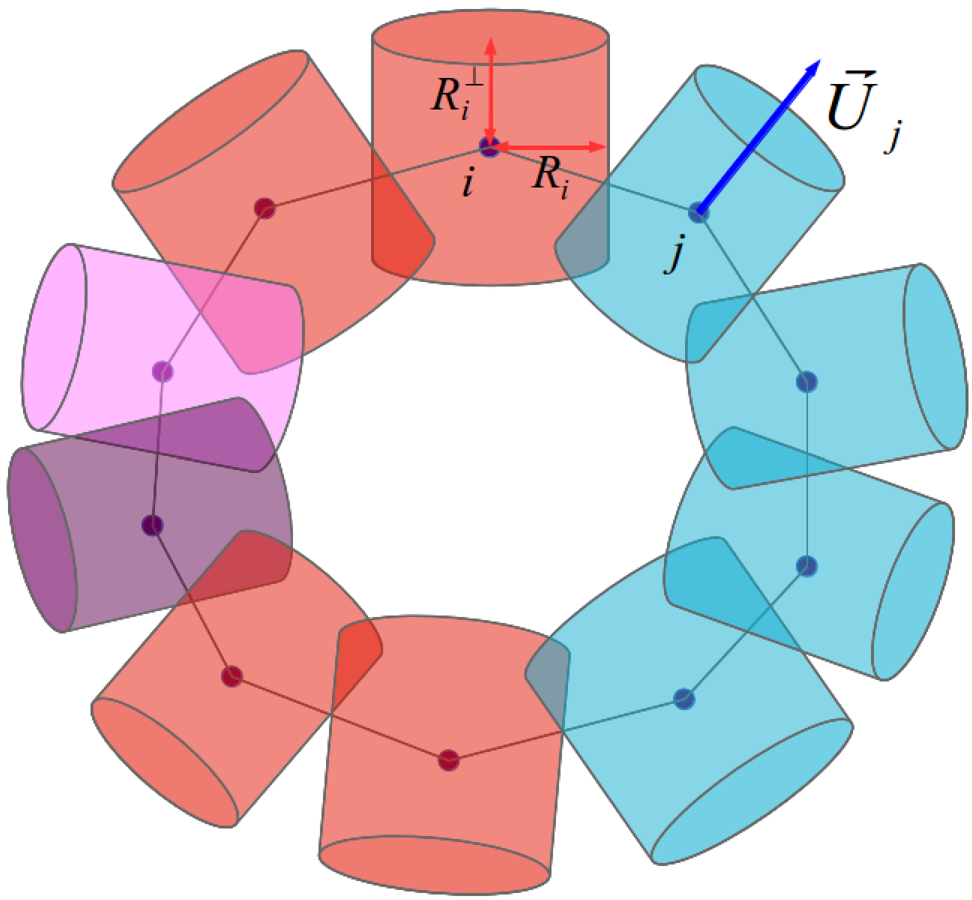

Supplementary Figure 12: Representation of cells by cylindrical particles. Each cell i or j is defined by a lateral radius R_i, a half-height $R_i^{\perp}$ and a normal vector $\vec{U}_i$ (R_j, $R_j^{\perp}$, $\vec{U}_j$ respectively).

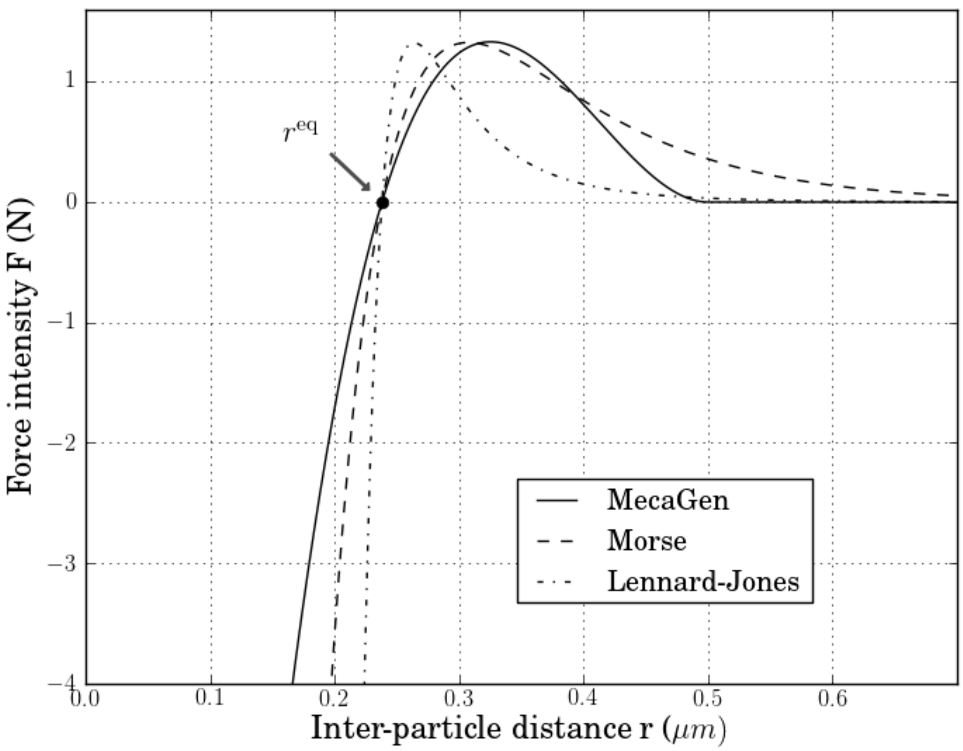

Supplementary Figure 13: Comparison of the attraction-repulsion force used here with alternative classical forces. The equilibrium distance is $r_{ij}^{\text{eq}} = c_{\text{eq}}(R_i + R_j)$ with $R_i = 0.1$ and $R_j = 0.15$. The solid line represents the attraction/repulsion potential $\vec{F}_{ij}^{\parallel}$ with $w_{\text{adh}} = w_{\text{rep}} = 500$. The dashed line represents a force derived from the Morse potential: $F_M(r) = 2Dke^{-k(r-r_{ij}^{\text{eq}})} - 2Dke^{-2k(r-r_{ij}^{\text{eq}})}$ with $D = 0.265$ and $k = 10$. The dot-dashed line is a force derived from the Lennard-Jones potential: $F_L(r) = -\frac{24\varepsilon}{\sigma}(2(\frac{r}{\sigma})^{-13} - (\frac{r}{\sigma})^{-7})$ with $\varepsilon = 0.117$ and $\sigma = 2^{-1/6} r_{ij}^{\text{eq}}$. Parameters were selected to make the equilibrium distance and maximum value of the three curves approximately match on the plot.

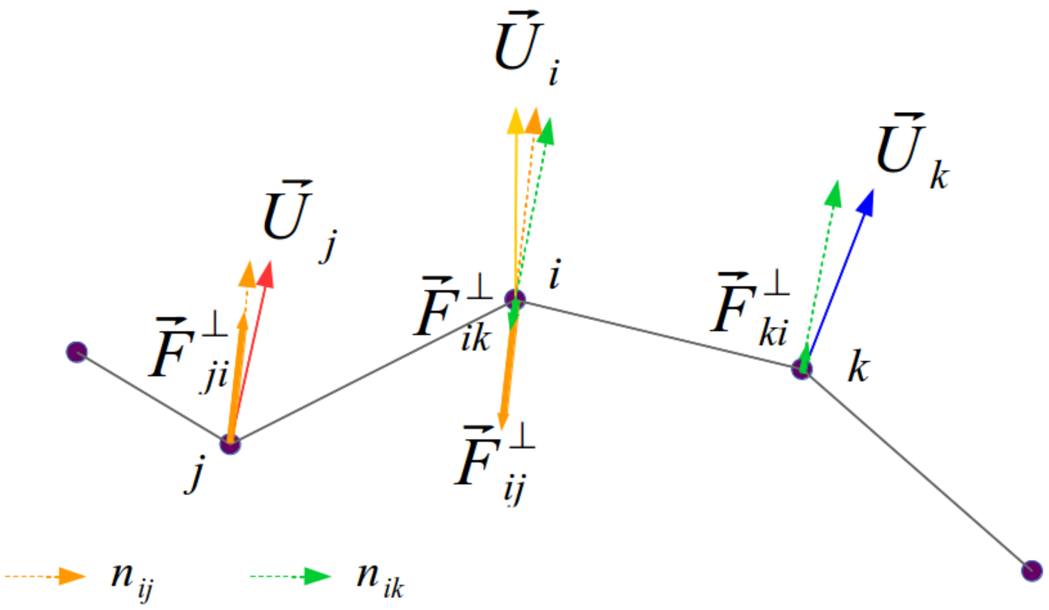

Supplementary Figure 14: Diagram of the planar rigidity force between neighbouring epithelial cells. Here, cells i, j and k are represented in 2D, with their respective normal axes $\vec{U}_i$ (yellow arrow), $\vec{U}_j$ (red arrow) and $\vec{U}_k$ (blue arrow). The planar rigidity forces exerted between neighbour cells i and j, and between i and k are aligned with the bisectors of the normal vectors $\vec{n}_{ij}$ (orange), and $\vec{n}_{ik}$ (green) respectively. Forces are represented by thicker arrows. The intensity of the force is less between i and k because the relative position vectors $\vec{u}_{ik}$ and $\vec{n}_{ik}$ are nearly orthogonal. As a result of these forces, neighbour cells are always pulled back to the lateral domain of each cell, thus maintaining the planarity of the tissue.

Specimen	Start	End	Duration	Δt	Objective	Size	Voxels
Embryo 1	3h45m	6h39m	2h54m	1m59s	63x/0.9NA	$246 \times 246 \times 90$	$0.48 \times 0.48 \times 0.96$
Embryo 2	5h45m	9h03m	3h18m	2m13s	63x/0.9NA	$246 \times 246 \times 100$	$0.48 \times 0.48 \times 0.96$
Embryo 3	4h20m	10h05m	5h45m	3m27s	20x/0.95NA	$280 \times 280 \times 86$	$0.55 \times 0.55 \times 1.09$
Embryo 4	5h00m	9h46m	4h46m	3m40s	20x/0.95NA	$269 \times 269 \times 88$	$0.53 \times 0.53 \times 1.05$
Embryo 5	4h20m	11h20m	7h00m	3m00s	20x/0.95NA	$266 \times 266 \times 82$	$0.52 \times 0.52 \times 1.05$

Supplementary Table 1: Technical details of data acquisition for the specimens of the cohort. Imaging starting and ending times in hours post fertilization. Timing prior rescaling. See Supplementary Fig. 2 for rescaled time. Volume in μm^3. Voxel size in μm^3. Objectives: Leica HCX APO L 63x/0.9NA U-V-I water and Olympus XLUMPFL 20x/0.95NA water. Estimated temperature at the level of the specimen 20C from temperature control in the room (18C).

Stage	SMic	LMic	Mac	Mes	Average Time in hpf
32 cells	4	4	8	16	4.5
60 cells	4	8	16	32	5.2
108 cells	4	8	32	64	5.8
216 cells	8	16	64	128	7.2

Supplementary Table 2: Average correspondence between hpf and stages corresponding to plateaus in the total number of cells as observed on Supplementary Fig. 2b

daughter $i \in \mathscr{L}_g$	mother $j \in \mathscr{L}_{g-1}$	sister $i_1 \in \mathscr{L}_g$	sister $i_2 \in \mathscr{L}_g$	R^2_{min}	R^2_{max}	#groups g such that $R^2 > 0.6$
x_i	m_j			3e-3	0.51	0 of 23
		x_{i_1}	$> x_{i_2}$	0.095	0.77	7 of 23
		a_{i_1}	$> a_{i_2}$	1.4e-3	0.88	5 of 38
a_i	$\bar{v}_j$			1.9e-4	0.43	0 of 38
		b_{i_1}	$> b_{i_2}$	0.02	0.81	8 of 38
b_i	$\bar{s}_j$			1.4e-5	0.39	0 of 38

Supplementary Table 3: Summary of the linear regressions for six pairs of features across 198 pairs of distributions in 61 groups of cells. Details can be found in Supplementary Note 2.2.3.

Cell group	$\mathscr{L}_g$ with $g = (e, n, k)$
Cardinality	$\lvert\mathscr{L}_g\rvert = 2\lvert\mathscr{L}_{g-1}\rvert = 2^{n-n'}\lvert\mathscr{L}_{g'}\rvert$ with $g-1 = (e, n-1, k)$ and $g' = (e, n' < n, k)$
Division time	$M_g = M_{g-1} + X_g = M_{g'} + \sum_{h=g'+1}^{g} X_h$ $M_g \sim \mathscr{N}(\mu_{M_g}, \sigma_{M_g})$ and $X_g \sim \mathscr{N}(\mu_{X_g}, \sigma_{X_g})$ $P(X_g \mid M_{g-1}) = P(X_g)$ $\mu_{M_g} = \mu_{M_{g'}} + \sum_{h=g'+1}^{g} \mu_{X_h}$ $\sigma_{M_g} = \sqrt{(\sigma_{M_{g'}})^2 + \sum_{h=g'+1}^{g} (\sigma_{X_h})^2}$
Average volume	$\overline{V}_g = \overline{V}_{g-1} A_g = \overline{V}_{g'} \left(\prod_{h=g'+1}^{g} A_h\right)$ $\overline{V}_g \sim \log\mathscr{N}(\mu_{\overline{V}_g}, \sigma_{\overline{V}_g})$ and $A_g \sim \log\mathscr{N}(\mu_{A_g}, \sigma_{A_g})$ $P(A_g \mid \overline{V}_{g-1}) = P(A_g)$ $\mu_{V_g} = \mu_{\overline{V}_{g'}} + \sum_{h=g'+1}^{g} \mu_{A_h}$ $\sigma_{V_g} = \sqrt{(\sigma_{\overline{V}_{g'}})^2 + \sum_{h=g'+1}^{g} (\sigma_{A_h})^2}$
Average surface area	$\overline{S}_g = \overline{S}_{g-1} B_g = \overline{S}_{g'} \left(\prod_{h=g'+1}^{g} B_h\right)$ $\overline{S}_g \sim \log\mathscr{N}(\mu_{\overline{S}_g}, \sigma_{\overline{S}_g})$ and $B_g \sim \log\mathscr{N}(\mu_{B_g}, \sigma_{B_g})$ $P(B_g \mid \overline{S}_{g-1}) = P(B_g)$ $\mu_{S_g} = \mu_{\overline{S}_{g'}} + \sum_{h=g'+1}^{g} \mu_{B_h}$ $\sigma_{S_g} = \sqrt{(\sigma_{\overline{S}_{g'}})^2 + \sum_{h=g'+1}^{g} (\sigma_{B_h})^2}$

Supplementary Table 4: Summary of the relationships between the different random variables in the multi-level probabilistic model. The random variables considered are the division time M, the cell cycle length X, the average volume $\overline{V}$, the average surface area $\overline{S}$, the daughter/mother volume ratio A, the daughter/mother surface area ratio B. See details in Supplementary Note 2.3.

Cells	$j \in \mathscr{L}_{g-1}$ and $i \in \mathscr{L}_g$<br>i is one of j's two daughters
Division time	$m_i = m_j + x_i$<br>m_j is a realization of M_{g-1}<br>x_i is a realization of X_g
Volume	$v_i(t) = a_i \overline{v}_j . \omega_g(u)$<br>$a_i$ is a realization of A_g<br>$\overline{v}_j$ is a realization of $\overline{V}_{g-1}$<br>ω_g is the deterministic volume microdynamics<br>u is the percentage of elapsed cycle length of cell i
Surface area	$s_i(t) = b_i \overline{s}_j . \phi_g(u)$<br>$b_i$ is a realization of B_g<br>$\overline{s}_j$ is a realization of $\overline{S}_{g-1}$<br>ϕ_g is the deterministic surface area microdynamics<br>u is the percentage of elapsed cycle length of cell i

Supplementary Table 5: Instantiation of the model independently in each branch and group of the cell lineage. The cell features considered are the division time m, the cell cycle length x, the average volume $\overline{v}$, the average surface area $\overline{s}$, the daughter/mother volume ratio a and the daughter/mother surface ratio b, corresponding random variables are denoted with uppercase letter (M, X, $\overline{V}$, $\overline{S}$, A, B respectively). The volume and surface area are modulated in time by volume micro dynamics ω and surface area micro dynamics ϕ. See details in Supplementary Note 2.3.

Parameter	Values Explored	Increment	Values Shown (Fig. 4, Supplementary Videos 4-7)
$w_{\text{adh,e}}$	[10, 1000]	+25	[10, 785]
$w_{\text{adh,o}}$	[10, 1000]	+25	[10, 785]
w_{rep}	100	–	100
c_{max}	2	–	2
k_{rig}	[6000, 16000]	+1000	10000
α_{gab}	[0.9, 1.3]	+0.02	1.0
λ_0	3000	–	3000

Supplementary Table 6: Exploration range and increments, with chosen values, for the parameters of the spatial model. $w_{\text{adh,e}}$ is the heterotypic adhesion coefficient, $w_{\text{adh,o}}$ is the homotypic adhesion coefficient, w_{rep} is the repulsion coefficient, c_{max} is used to define the maximum cell deformation in the surface plane, these four coefficients are involved in the computation of the attraction-repulsion force. k_{rig} is the rigidity coefficient used to compute the planar rigidity force. α_{gab} is a coefficient used to define the neighbourhood of each cell through a 3D generalization of the Gabriel criterion. λ_0 is used to compute the damping coefficient, required to compute the equation of motion. The full description of the spatial model can be found in Supplementary Note 3.1

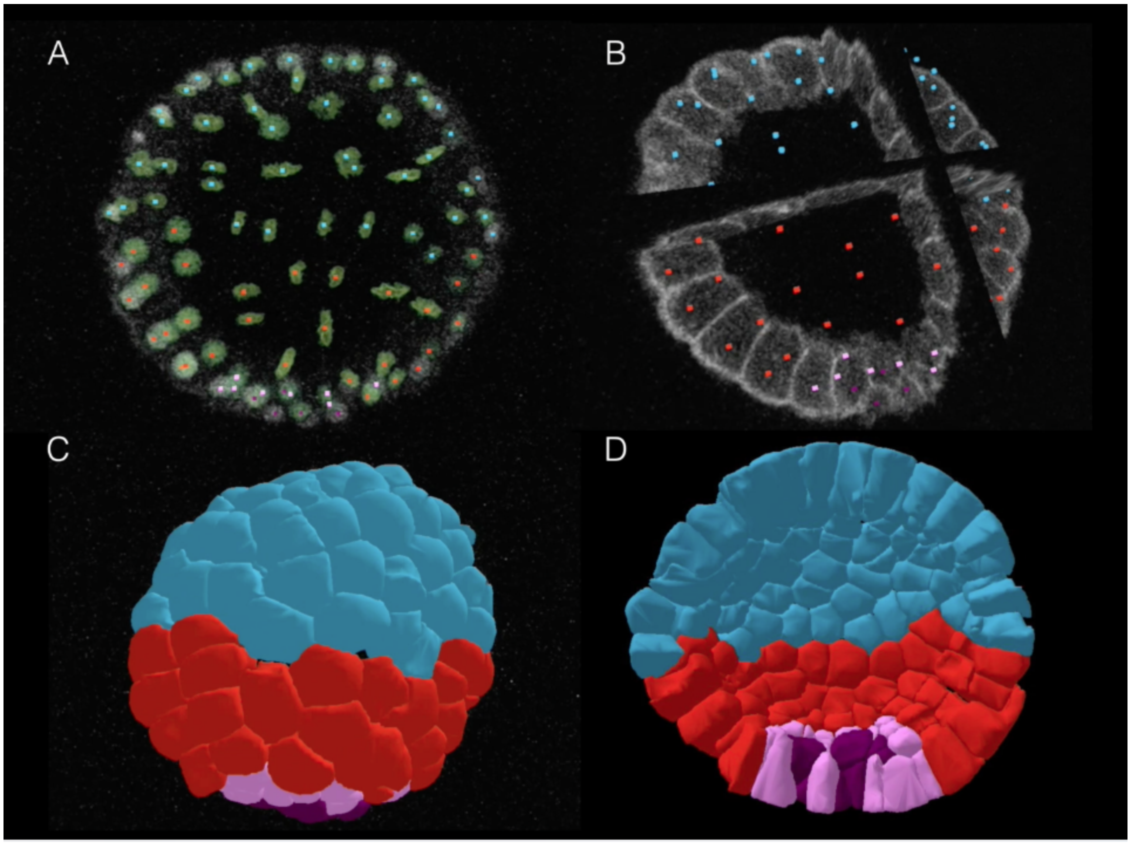

Supplementary Video 1: Visualization of reconstructed embryo 3 visualized from 4.56 hpf (32-cell stage) to 8.65 hpf (hatching blastula) with Mov-IT software: A) Raw nuclei, coloured dots are detected nuclear centres (mesomeres in blue, macromeres in red, large micromeres in pink, small micromeres in purple). Volume rendering of raw images from two-photon laser scanning microscopy with nuclear staining by H2B-mCherry. Note that at the onset of imaging, membrane signal is leaking in the nucleus channel. Lateral view animal to the top. B) Raw membranes, the coloured dots are the same as in A). Three orthoslices (xy imaging plane, xz and xy planes orthogonal to the imaging plane) showing raw images from two-photon laser scanning microscopy with membrane staining by farnesylated eGFP. The animal orientation can be inferred from the organization of the cell populations. C) Surface rendering of segmented cell membranes. (mesomeres in blue, macromeres in red, large micromeres in pink, small micromeres in purple). Lateral view animal to the top. D) Same as C). The embryo is cut along a radial plane and shown from the blastocoel.

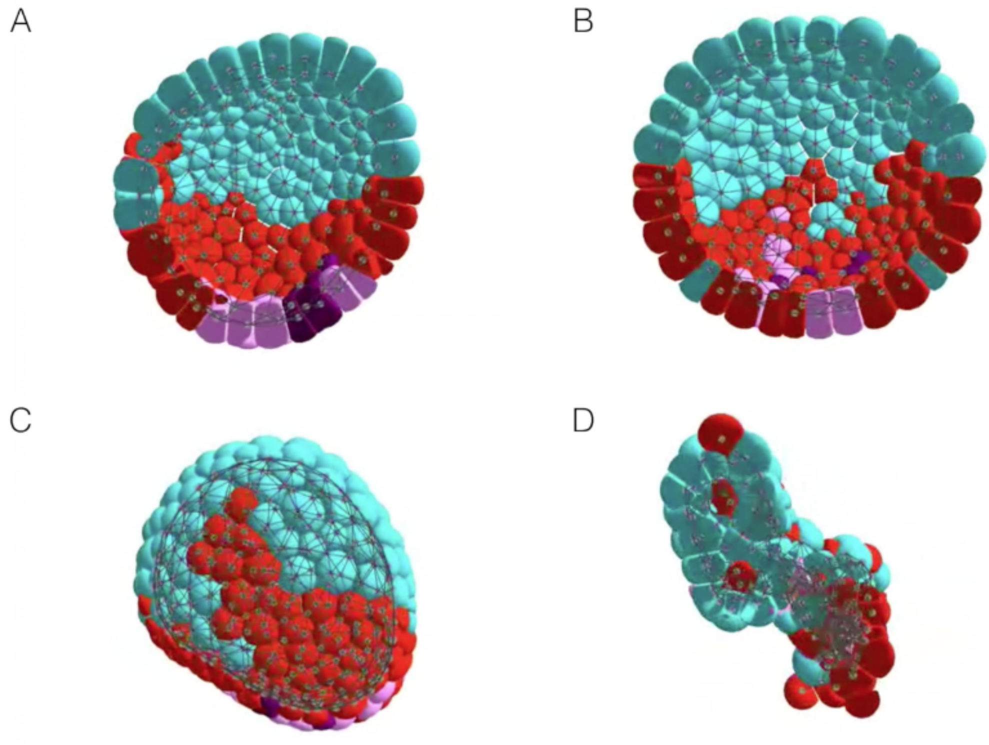

Supplementary Video 2: Modelling of sea urchin prototypical early development with the MecaGen platform and simulation with four different sets of mechanical parameters: A) Simulation of a monolayered, spherical embryo with realistic cell population borders. This specimen falls into the best-fit domain. It was obtained with the following parameters: $w_{\mathrm{adh,o}} = 600$, $w_{\mathrm{adh,e}} = 110$, $w_{\mathrm{rep}} = 100$, $k_{\mathrm{rig}} = 10000$, $\alpha_{\mathrm{gab}} = 1$, $\lambda_0 = 3000$, and $c_{\max} = 2$. The embryo is cut along a radial plane and shown from the blastocoel. Black lines materialize cell-cell contacts. B) Simulation of a monolayered, spherical embryo with nonrealistic cell population borders. Although the global shape is correct, this simulated embryo shows cell population borders that differ from the live specimens with nonrealistic cell mixing (pink area in Fig. 4c). It was obtained with the following parameters: $w_{\mathrm{adh,o}} = 100$, $w_{\mathrm{adh,e}} = 200$, $w_{\mathrm{rep}} = 100$, $k_{\mathrm{rig}} = 10000$, $\alpha_{\mathrm{gab}} = 1$, $\lambda_0 = 3000$, and $c_{\max} = 2$. The embryo is cut along a sagittal plane and shown from the blastocoel. Black lines materialize cell-cell contacts. C) Simulation of a monolayered, aspherical embryo with nonrealistic cell population borders. The simulated embryo still displays an epithelial monolayer but not the appropriate sphericity (in addition to cell population borders that differ from the live specimens with nonrealistic cell mixing). Since the embryo collapsed, it was not possible to define a radial plane. It is displayed *in toto* from the outside with the whole scaffold of cell-cell contacts (black lines) and was obtained with the following parameters: $w_{\mathrm{adh,o}} = 400$, $w_{\mathrm{adh,e}} = 220$, $w_{\mathrm{rep}} = 100$, $k_{\mathrm{rig}} = 10000$, $\alpha_{\mathrm{gab}} = 1$, $\lambda_0 = 3000$, and $c_{\max} = 2$. D) Simulation of a multilayered, aspherical embryo with nonrealistic cell population borders. This simulated specimen cumulates all three defects and crumpled upon itself. It was not possible to define a radial plane in this case. The embryo is cut along a longitudinal plane and shown from the blastocoel. Black lines materialize cell-cell contacts. It was obtained with the following parameters: $w_{\mathrm{adh,o}} = 700$, $w_{\mathrm{adh,e}} = 700$, $w_{\mathrm{rep}} = 100$, $k_{\mathrm{rig}} = 10000$, $\alpha_{\mathrm{gab}} = 1$, $\lambda_0 = 3000$, and $c_{\max} = 2$.

Supplementary Note 1 Digital embryo reconstruction

As anticipated in [1], we provide the first complete digital reconstruction of the sea urchin embryo's early development, comprising the position and shape of every cell over time. The methods and tools used here are part of the BioEmergences platform developed in our laboratory [2]. Cell positions were detected at the local maxima of cell nucleus images filtered by a difference of Gaussians (DoG), while cell shapes were reconstructed from cell membrane images using the "subjective surface" algorithm (SubSurf) [3], following earlier results [4]. To reconstruct cell trajectories, cell movements were tracked by minimizing a heuristic functional with simulated annealing (SimAnn) [5]. The choice of parameters for DoG filtering was supervised, and all cell positions and trajectories were manually checked and validated through our dedicated Mov-IT software interface [6, 2].

Supplementary Note 1.1 Nucleus center detection

In order to detect cell positions, we assumed that cells could be identified by the spatial coordinates of nuclear centres. Raw imaging data was preprocessed to simultaneously smooth images and preserve only the spatial information relevant to the size of cell nuclei. Filtering was carried out by a convolution of the original image with two Gaussian functions G_1 and G_2 of variances $\sigma_1 < \sigma_2$, and calculating the difference $G_1 - G_2$. The output was renormalized to be in the range [0,1]. Since a few spurious regions could still be present in the image, we applied a simple threshold θ, setting all lower values to zero. Finally, cell positions were identified with the local maxima of the resulting image. Considering the changes in size of cell nuclei during the acquisition and the variations in intensity over time and among different datasets, we opted for a supervised choice of parameters. The algorithm was run for $\sigma_1 = 1.2, 1.4, ..., 2.6$ µm, $\sigma_2 = 12, 14, 16$ µm and $\theta = 0.01, 0.02, ..., 0.09$. Our custom interface Mov-IT made it possible to superimpose raw data on the detected cell positions for various parameter values. Parameters were manually chosen at a few time steps selected along each time lapse, then intermediate values were interpolated linearly from them.

Supplementary Note 1.2 Cell tracking

The objective of cell tracking is to detect the invariant identity of each cell by relating its positions at two consecutive time steps, from its birth at the last mitosis to its disappearance at the next mitosis. Starting with the initial cell population, temporal links create together a *lineage tree* whose branches split at cell divisions. Considering that sea urchin embryos were immobilized and cells did not move fast at the observed early stages of development, our tracking strategy essentially relied on a nearest-neighbour criterion to link detected nuclear centres. First, tree branches were constructed by connecting nearest cells on consecutive images when that proximity criterion was reciprocal. Then, mitoses were detected by linking cells left over without a predecessor to their closest neighbour at the previous time step. Finally, the resulting lineage tree was refined via minimization of a functional $E(f)$. This energy term assumes that the embryo behaves like an elastic mass, therefore cells that are neighbours at time t remain close to each other at time $t+1$:

$$E(f) = \sum_{(i,j)} \|(\vec{r}_j - \vec{r}_i) - (\vec{r}_{f(j)} - \vec{r}_{f(i)})\|^2, \qquad 1$$

where i, j represent the cells, f is the link between cells at time t and cells at time $t+1$, and $\vec{r}_i = (x_i, y_i, z_i)$ is the cell position. The minimization of the functional was performed by a simulated annealing algorithm [5], searching for the lowest-energy configuration by randomly changing a link, removing/adding a link or removing/adding a cell. Finally, we refined the automated tracking by removing cells with a very short cell cycle (e.g. a few time steps), as they were most likely to be false positives.

Supplementary Note 1.3 Digital specimen validation and correction

All cell positions and trajectories were checked by at least two experts through visual inspection comparing the digital reconstruction with the raw data. All detectable errors (false positive and false negative nuclei, false links, missing links) were corrected to build error-free lineages—hereafter called “gold standard”. All five lineage trees were fully validated for all five datasets in this manner. Moreover, each cell was unambiguously identified by a unique ID in our database. Both operations were a prerequisite to perform the next steps comprising cell segmentation, which uses cell positions for initialization and cell IDs for labelling, and data analysis.

Supplementary Note 1.4 Cell segmentation

Three-dimensional segmentation of *in vivo* imaging presents a difficult challenge, especially since fast and deep two-photon laser scanning microscopy produces a low signal-to-noise ratio and incomplete membrane contours, thus dedicated methods have to be used for this task. To this purpose, cell shapes were obtained by applying a generalized version of the SubSurf method [4] for its ability to reconstruct missing boundaries, making it particularly suitable for this type of segmentation. To remove the noise and smooth the images while faithfully preserving edge information, the data was first filtered by geodesic mean curvature flow (GMCF) [7]. Segmentation of cell membranes was then performed on the filtered data using the manually validated cell positions as starting points. Each membrane was segmented independently on a region of interest located around the initialization points and ranging from $\pm 25\mu\text{m}$ to $\pm 16\mu\text{m}$ in every direction depending on the number of cells in the processed volume. Finally, the whole embryo shape was recomposed from the union of the segmented cells and each region labelled according to the ID of the corresponding cell centre. Information was exchanged between adjacent membranes to avoid overlapping. In particular, superimposed regions were split depending on the distance from the nearest cell centre and the ID of the obtained subregions set accordingly. Segmentation results were exported in two formats: voxel-based image data to support the calculation of cell volumes and the detection of neighbour cells; and triangulated surfaces to support the calculation of cell surface areas.

The GMCF and generalized SubSurf methods are briefly summarized in the next paragraphs. Numerical implementation of both filtering and segmentation algorithms was performed via finite difference schemes [8, 3]. Further details about the methods employed can be found in [7] and [4].

Supplementary Note 1.4.1 Image filtering

We model the 3D input image by a real positive function $I^0(x,y,z)$, in some spatial rectangular domain $\Omega \subset \mathbb{R}^3$, and its low-level local features by an edge indicator function g (see below). According to GMCF, a smooth-edge enhanced version of the original image can be obtained by extending the image function in time, creating a family of “level sets” $I(x,y,z,t)$, and solving the following partial differential equation:

$$\partial_t I = |\nabla I| \, \nabla \cdot \left(g(|\nabla G_\sigma * I|) \frac{\nabla I}{|\nabla I|} \right) \qquad 2$$

where G_σ is a Gaussian kernel with a small variance σ. This is accompanied by the initial condition $I(x,y,z,0) = I^0(x,y,z)$ and a null Neumann boundary condition (i.e. zero gradients) on the domain edges $\partial\Omega$. Selecting a final time $t = T$, the solution $I(x,y,z,T)$ represents the filtered image I_f. In the model, the mean curvature motion of the level sets of function I is determined by the edge indicator function $g(s) = 1/(1+Ks^2)$, $K \geq 0$, applied to the image intensity gradient pre-smoothed by G_σ.

Supplementary Note 1.4.2 Image segmentation

In the 3D case, the SubSurf method consists of minimizing the volume of a 3D manifold S embedded in a 4D Riemannian space with a metric constructed on the image features [4]. We consider the filtered image I_f and its low-level local features given by the same edge detector function $g_f(x,y,z) = g(I_f(x,y,z))$ as for the GMCF. Let also the manifold be defined as $S = (x,y,z,\Phi)$, where $\Phi(x,y,z)$ is a positive distance function defined in the image domain Ω. The generalized version of the SubSurf method leading to the minimization of the volume of S can be expressed as a function of Φ as follows:

$$\begin{cases} \partial_t \Phi = \mu \, g_f \, H \, |\nabla\Phi|_\varepsilon + \nu \, \nabla g_f \cdot \nabla\Phi & \text{in } \Omega \times]0,\tau[\\ \Phi(x,y,z,t) = \min(\Phi_0) & \text{in } \partial\Omega \times]0,\tau[\\ \Phi(x,y,z,0) = \Phi_0 & \text{for } (x,y,z) \in \Omega, \end{cases} \quad 3$$

where μ and ν are weight coefficients and τ is the value of the scale parameter t which corresponds to the steady-state condition of the first equation. The notation $|\nabla\Phi|_\varepsilon = \sqrt{\varepsilon + |\nabla\Phi|^2}$ represents a regularization of $|\nabla\Phi|$ [9] and H reads:

$$H = \frac{(\varepsilon + \Phi_x^2 + \Phi_y^2)\Phi_{zz} + (\varepsilon + \Phi_x^2 + \Phi_z^2)\Phi_{yy} + (\varepsilon + \Phi_y^2 + \Phi_z^2)\Phi_{xx}}{(\varepsilon + \Phi_x^2 + \Phi_y^2 + \Phi_z^2)^{3/2}} - 2\frac{\Phi_x\Phi_z\Phi_{xz} + \Phi_x\Phi_y\Phi_{xy} + \Phi_y\Phi_z\Phi_{yz}}{(\varepsilon + \Phi_x^2 + \Phi_y^2 + \Phi_z^2)^{3/2}}. \quad 4$$

where subscripts are shortcuts for derivatives: $\Phi_x = \partial\Phi/\partial x$, $\Phi_{xx} = \partial^2\Phi/\partial x^2$, etc.

The first term on the right-hand side of equation 3 (top row) represents a mean curvature flow weighted by the edge indicator g_f. The second term attracts the level sets of Φ in the direction of the image edges, given by the velocity field $-\nabla g_f$. Different behaviors can be identified locally in the image regions according to one of these flows, and missing boundaries (the so-called "subjective contours"), which are a continuation of existing edge fragments, are completed by geodesics. The use of different weights between the regularization and the advective term ($\nu > \mu$) facilitates the control of the dynamic process, while the parameter ε is used to shift the model from the mean curvature flow of the level sets ($\varepsilon = 0$) to the mean curvature flow of the graph ($\varepsilon = 1$).

Supplementary Note 2 Multi-level probabilistic model of the cell lineage

Supplementary Note 2.1 Multi-level measures and rescaling

Supplementary Note 2.1.1 Individual cell features

The digital reconstruction of entire sea urchin specimens allowed us to extract a variety of features at the level of individual cells (Supplementary Note 1) [4]. For any cell i, we defined the following quantities (Supplementary Fig. 1):

- its *cell cycle length*: x_i, corresponding to the time between two consecutive divisions
- a *mitosis time*: m_i, denoting the moment at which the cell i divides into two daughter cells
- its *current volume*: $v_i(t)$, with the *average volume* over its cell cycle length (using discrete time steps):

$$\overline{v}_i = \frac{1}{x_i}\int_{m_i - x_i}^{m_i} v_i(t)dt \ \simeq \ \frac{1}{x_i}\sum_{t=m_i-x_i}^{m_i-1} v_i(t) \qquad 5$$

- its *current surface area*: $s_i(t)$, with the *average surface area* over its cell cycle length:

$$\overline{s}_i \simeq \frac{1}{x_i}\sum_{t=m_i-x_i}^{m_i-1} s_i(t) \qquad 6$$

- a *generation number*: n_i, denoting the number of past divisions on cell i's lineage branch, which includes itself and all its ancestors since fertilization
- a *cell type*: k_i, taking one of four possible values: 1 for mesomeres (Mes), 2 for macromeres (Mac), 3 for large micromeres (LMic), and 4 for small micromeres (SMic) [10] (Supplementary Fig. 1b)
- its *current degree*: $d_i(t)$, equal to the number of neighbours of cell i at time t, assuming that the spatial distribution of cells can be represented by an undirected graph of cellular contacts, in which cells correspond to nodes and cell-cell interactions to edges [11, 12].

Each cell i also has a mother identified by j (a shorthand notation for the function $j(i)$ mapping any cell index to its mother's index), which is used in the definition of two additional features:

- a daughter/mother *average volume ratio*: $a_i = \overline{v}_i/\overline{v}_j$
- a daughter/mother *average surface ratio*: $b_i = \overline{s}_i/\overline{s}_j$

and three relationships:

- the mitosis time of a cell is equal to the sum of its cell cycle length and the mitosis time of its mother: $m_i = m_j + x_i$
- the generation is incremented by one at each division: $n_i = n_j + 1$
- starting from the 32-cell stage, each cell type is conserved across divisions throughout the period considered here (formation of the blastula): $k_i = k_j$.

Supplementary Note 2.1.2 Cell populations and global state variables

The *cell lineage*, denoted by $\mathscr{L}$, is the set of all cells, past and present, that the embryo contained during a given period of time. From there, various subsets were defined:

- the *current embryo*, the set of all cells alive at time t: $\mathscr{L}(t) = \{i \in \mathscr{L} \mid m_i - x_i \leq t < m_i\}$
- *cell populations*, the sets of cells of a given type k across all generations: $\mathscr{L}^k = \{i \in \mathscr{L} \mid k_i = k\}$
- *current cell populations*, the cell population at time t: $\mathscr{L}^k(t) = \{i \in \mathscr{L}(t) \mid k_i = k\}$.

At the level of the entire embryo, and at each time step t, the global measures were:

- the *number of cells*, cardinality of the current embryo: $N(t) = |\mathscr{L}(t)|$
- the *total cellular volume*, sum of all individual cell volumes: $W(t) = \sum_{i \in \mathscr{L}(t)} v_i(t)$
- the *total cellular surface area*, sum of individual cell surface areas: $Z(t) = \sum_{i \in \mathscr{L}(t)} s_i(t)$
- the *total number of contacts*, sum of the local degrees: $C(t) = \sum_{i \in \mathscr{L}(t)} d_i(t)$

These quantities were also calculated inside each cell population k, replacing $\mathscr{L}(t)$ by $\mathscr{L}^k(t)$ in the above definitions and using the notations $N^k(t)$, $W^k(t)$, $Z^k(t)$, and $C^k(t)$.

Supplementary Note 2.1.3 Rescaled embryo dynamics

The number of cells, total cellular volume, and total cellular surface area were measured empirically in each embryo of the cohort $\mathscr{E}$, indexed by $e \in [1,5]$ and denoted $N_e(t)$, $N_e^k(t)$, and so on. The evolution over time of these variables was plotted concurrently (Supplementary Fig. 2a,d,g). We observed that the patterns of evolution across the various specimens were similar but did not overlap: some were globally faster, some slower; some were larger, other smaller. To filter out this variability and proceed to further interindividual comparison, we applied temporal and spatial rescaling functions.

Temporal rescaling For each observed cell number $N_e(t)$, we considered its inverse function $t_e(N)$ equal to the time at which embryo e contained a given number N of cells (symmetric of Supplementary Fig. 2a). Since $N_e(t)$ is a monotonically increasing function (no cell death during the period considered), so is $t_e(N)$. However, because of the relative synchrony among cell cycles, N_e can remain constant for several time steps (plateaus in Supplementary Fig. 2a). Therefore, to obtain a single-valued function, we defined $t_e(N) = \min\{t \mid N_e(t) = N\}$ over the size interval of e, $[N_{e,\min}, N_{e,\max}]$ (if N was not part of the observed discrete values, $t_e(N)$ was interpolated). Then, to minimize the discrepancy between the observed time of an embryo, $t_e(N)$, and the mean time of the five specimens, $\langle t\rangle(N)$, an affine transform was performed on the time axis using a cost function:

$$F_e(\alpha,\beta) = \sum_{N=N_{e,\min}}^{N_{e,\max}} (\alpha t_e(N) + \beta - \langle t\rangle(N))^2 \quad \text{with} \quad \langle t\rangle(N) = \frac{1}{|\mathscr{E}_N|} \sum_{e \in \mathscr{E}_N} t_e(N), \tag{7}$$

where $\mathscr{E}_N$ is the sublist of embryos e such that $N \in [N_{e,\min}, N_{e,\max}]$ (including by interpolation). For each embryo, the parameters adopted for the transform satisfied $(\alpha_e, \beta_e) = \arg\min F_e(\alpha, \beta)$. Finally, taking the inverse again, we obtained five rescaled total numbers $N_e(\alpha_e t + \beta_e)$ and four groups of five rescaled field numbers $N_e^k(\alpha_e t + \beta_e)$, which are plotted in Supplementary Fig. 2b. The five parameter pairs (α_e, β_e) are shown in Supplementary Fig. 2c.

Spatial rescaling Based on the same temporal rescaling, a linear transform was also performed along the spatial dimensions to minimize the discrepancy between the temporally rescaled cellular volume of an embryo, $W_e(\alpha_e t + \beta_e)$ and its mean value over all specimens:

$$G_e(\gamma) = \sum_{t=(t_{e,\min}-\beta_e)/\alpha_e}^{(t_{e,\max}-\beta_e)/\alpha_e} (\gamma^3 W_e(\alpha_e t + \beta_e) - \langle W \rangle (t))^2 \quad \text{with} \quad \langle W \rangle (t) = \frac{1}{|\mathscr{E}_t|} \sum_{e \in \mathscr{E}_t} W_e(\alpha_e t + \beta_e), \tag{8}$$

where $\mathscr{E}_t$ is the sublist of embryos e such that $(\alpha_e t + \beta_e) \in [t_{e,\min}, t_{e,\max}]$ (including by interpolation). For each embryo, $\gamma_e = \arg\min G_e(\gamma)$ was then defined as the optimal coefficient. The original volumes $W_e(t)$ are plotted in Supplementary Fig. 2d. The five rescaled total volumes $(\gamma_e)^3 W_e(\alpha_e t + \beta_e)$ and four groups of five rescaled field volumes $(\gamma_e)^3 W_e^k(\alpha_e t + \beta_e)$ each are plotted in Supplementary Fig. 2e, while coefficients γ_e are shown in Supplementary Fig. 2f. Finally, we used the same coefficients squared for the rescaled cellular surface areas, $(\gamma_e)^2 Z_e(\alpha_e t + \beta_e)$ and $(\gamma_e)^2 Z_e^k(\alpha_e t + \beta_e)$ (Supplementary Fig. 2h; the original curves $Z_e(t)$ are shown in Supplementary Fig. 2g).

neighbourhoods The total numbers of contacts in each embryo and each cell population did not require an extra spatial transform to highlight interindividual similarities. After carrying over the temporal rescaling parameters found above, the curves $C_e(\alpha_e t + \beta_e)$ and $C_e^k(\alpha_e t + \beta_e)$ already presented a high degree of overlap among embryos $e \in [1,5]$ (Supplementary Fig. 2i). This is consistent with the fact that neighbourhood relationships are topological features, thus independent from metric deformations.

Supplementary Note 2.1.4 Intermediate cell groups

Symmetries in the embryo, such as the rotational symmetry around the animal-vegetal (AV) axis, prevent the identification and matching of individual cells from one specimen to another. Unique identification of cells based on their morphological characteristics cannot be done without ambiguity either. To overcome these issues, a generic *coarse-grained level* of observation was needed. We chose here to define new subsets of $\mathscr{L}$ (adding to the list of Supplementary Note 2.1.2):

- *generational cell groups*, or simply "cell groups", the subsets of generation-n cells of a given type k: $\mathscr{L}^{n,k} = \{i \in \mathscr{L} \mid n_i = n \;\&\; k_i = k\}$.

Note that $\mathscr{L}^{n,k}$ is not the same as $\mathscr{L}^k(t)$: the latter is a snapshot of cell population k at time t and therefore may contain a mix of generations if some cells have divided more frequently than others. Conversely, cells in the former group may have to be taken at different times. These sets offer two useful viewpoints on the embryo, one focusing on its global state, the other on cell statistics. In any case, the greater n and t, the more distant these two sets are likely to be.

At this intermediate scale, it became possible to identify and map cell groups $\mathscr{L}_e^{n,k}$ across embryos. At the lower level, cells belonging to the same group were expected to display similar individual features. At the sixth generation, each embryo e contained a total of 32 cells composed of 16 Mes, 8 Mac, 4 LMic, and 4 SMic cells, which can be written: $|\mathscr{L}_e^{6,1}| = 16$, $|\mathscr{L}_e^{6,2}| = 8$, $|\mathscr{L}_e^{6,3}| = |\mathscr{L}_e^{6,4}| = 4$ for all $e \in [1,5]$. Since each cell gives rise to two daughter cells at each cycle and there is no cell death, the number of cells of a given type continued doubling, i.e. $|\mathscr{L}_e^{n,k}| = 2^{n-6} |\mathscr{L}_e^{6,k}|$ for $n \geq 6$ during the period of interest.

The six cell features considered here are the cell cycle length x_i, mitosis time m_i, average cell volume $\overline{v}_i$, average cell surface area $\overline{s}_i$, and the daughter/mother ratios of average volume a_i and surface area b_i. The dispersion of these features observed in each group $\mathscr{L}_e^{n,k}$ of the empirical data is represented by *distributions*, modelled by sequences of random variables. For example, $(x_1, x_2, ..., x_m)$ denotes the sequence of the values that the first feature, cell cycle length, takes in the m cells of a given group $\mathscr{L}_e^{n,k}$. Each x_i is the realization of a random variable X_i pertaining to cell i. Cells being indistinguishable within the same group, their order

in the sequence is irrelevant. This property is referred to as "exchangeability" of the sequence of random variables and is formalized as follows: for any permutation π of the indices $[1,m]$, the joint probability distribution of these variables is the same:

$$P(X_{\pi(1)}, X_{\pi(2)}, ..., X_{\pi(m)}) = P(X_1, X_2, ..., X_m). \qquad 9$$

This property leads to de Finetti's theorem, which can be summarized by saying that an infinite sequence of exchangeable random variables is a "mixture" of independent and identically distributed (i.i.d.) sequences [13, 14]. As for a finite exchangeable sequence, it can also be approximated by a mixture of i.i.d. sequences [15]. One consequence of this theorem is that the following empirical measure P_{m,X_g} constitutes a summary statistic for the probability density P of the X sequence:

$$P_{m,X_g}(I) = \frac{1}{m}\sum_{i=1}^{m} \delta_{X_i}(I), \quad \text{with} \quad \delta_x(I) = 1 \ \text{ if } \ \times \in I \ \text{ and } \ \delta_x(I) = 0 \ \text{ if } \ \times \notin I, \qquad 10$$

where I is an interval of values or union of intervals (i.e. an element of the "σ-algebra" on $\mathbb{R}$) and δ_x is the Dirac measure. If m could increase to ∞, P_{m,X_g} would converge asymptotically to the underlying theoretical probability distribution P of the sequence of exchangeable random variables.

The motivation of the probabilistic model presented here and in the next section was to provide an idealized representation of the dynamics of multicellular development relating the distributions observed in each cell group with the global dynamics of the cell lineage. To simplify notations, let us define an embryo-specific cell group index $g = (e, n, k)$ so that $\mathscr{L}_e^{n,k}$ can be equivalently written $\mathscr{L}_g$. With this, our goal was the following:

- link the distribution in each group $\mathscr{L}_g$ of cell cycle lengths: $X_g \sim \{x_i \mid i \in \mathscr{L}_g\}$ and the distribution of mitosis times: $M_g \sim \{m_i \mid i \in \mathscr{L}_g\}$ to the evolution of the total number of cells in the embryo $N_e(t)$
- link the distribution in each group $\mathscr{L}_g$ of average volumes $\overline{V}_g$ and the distribution of daughter/mother volume ratios A_g to the evolution of the total cellular volume $W_e(t)$
- link the distribution in each group $\mathscr{L}_g$ of average surface areas $\overline{S}_g$ and the distribution of daughter/mother surface ratios B_g to the evolution of the total cellular surface area $Z_e(t)$.

Supplementary Note 2.2 Observation and approximation of multi-level statistics

From the empirical distributions of individual cell features, we derived parameterized representations approximating their probability distributions in each cell group. Combining cell features along the cell lineage required considering simple models of inheritance. As explained below, we calculated correlations based on an assumption of linearity in intercellular dependencies and, obtaining low values, concluded that the individual features of a cell were largely independent from its mother's or sister's features. This independence was then taken as a founding hypothesis of our model.

Supplementary Note 2.2.1 Estimation of cell feature distributions in cell groups

To interpret the empirical feature distributions, capture their evolution and predict the developmental dynamics, it was best to describe them in terms of parametric models. First, however, because of the finite and relatively short duration of the period of observation, some groups $\mathscr{L}_g$ were incompletely represented in the dataset. Therefore, a distribution of cell features in a group was considered to be significant only if the number of observed cells constituted at least 95% of the full cardinality $|\mathscr{L}_g|$ calculated above. Moreover, certain features, such as the volume v_i and surface area s_i, required the observation of their evolution during

whole cell cycles. Incomplete cell cycles were taken into account only if the mean period of observation of individual cell dynamics in a group was at least 20 min. Based on these criteria, we retained 252 distributions of individual cell features across all cell groups $\mathscr{L}_g$ (corresponding to a rough average of 4×3 significant distribution-generation pairs in each one of the 5×4 embryo-cell types).

Graphical assessment of these histograms led us to categorize them into two major types: *normal distributions* (i.e. Gaussian curves) for cell cycle lengths x_i and mitosis times m_i; and *log-normal distributions* for average cell volumes $\overline{v}_i$, average cell surface areas $\overline{s}_i$, daughter/mother volume ratios a_i and surface area ratios b_i (where a random variable X is said to be log-normally distributed if the random variable $Y = \log X$ is normally distributed).

We performed a chi-squared goodness-of-fit test on each distribution to validate our assessment. This test evaluates the proximity of the empirical frequency with the theoretical one, i.e. whether the random variables are i.i.d. under a normal distribution or a log-normal distribution. To this aim, we first calculated the *empirical mean and standard deviation* of each random variable in each cell group $\mathscr{L}_g$ using a classical maximum likelihood estimation [16]. For example, in the case of cell cycle lengths X_g these two quantities are

$$\begin{cases} \mu_{X_g} \approx \widetilde{\mu}_{X_g} = \frac{1}{N_g} \sum\limits_{i \in \mathscr{L}_g} x_i \\ \sigma_{X_g} \approx \widetilde{\sigma}_{X_g} = \sqrt{\frac{1}{N_g - 1} \sum\limits_{i \in \mathscr{L}_g} (x_i - \widetilde{\mu}_{X_g})^2} \end{cases} \quad 11$$

where $N_g = |\mathscr{L}_g|$. The same formulas were applied to variables M_g, $\log \overline{V}_g$, $\log \overline{S}_g$, $\log A_g$, or $\log B_g$, for each one of the 252 available distributions, yielding parameters $(\widetilde{\mu}_{M_g}, \widetilde{\sigma}_{M_g}), \ldots, (\widetilde{\mu}_{B_g}, \widetilde{\sigma}_{B_g})$. All 252 (μ, σ) pairs are plotted in Supplementary Fig. 3.

Then, based on the empirical mean and standard deviation, our categorization hypothesis was challenged in each distribution by calculating a p-value corresponding to the probability to find a good fit with a normal or log-normal curve. Taking again X_g as an example, the distribution of cell cycle lengths $\{x_1, x_2, \ldots, x_m\}$ over the m cells of group $\mathscr{L}_g$ was categorized into a fixed number l of discrete bins, $(\hat{x}_1, \hat{x}_2, \ldots, \hat{x}_l)$, where $\hat{x}_1 = \min\{x_i\}$ and $\hat{x}_{h+1} - \hat{x}_h = \Delta\hat{x} = (\max\{x_i\} - \min\{x_i\})/l$ for all $h \in [1, l-1]$. In this histogram, the *observed* distribution corresponds to the number of values falling in each bin and was given by the empirical measure $P_{m,X_g}(I_h)$ (equation 10), where $I_h = [\hat{x}_h, \hat{x}_h + \Delta\hat{x})$ and I_l is closed, while the *expected* distribution was given by $P_{m,\widetilde{X}_g}(I_h)$, where $\widetilde{X}_g$ is the theoretical normal distribution calculated from $(\widetilde{\mu}_{X_g}, \widetilde{\sigma}_{X_g})$. The chi-squared statistic computes the discrepancy between both distributions as follows:

$$\chi_g^2 = \sum_{h=1}^{l} \frac{(P_{m,X_g}(I_h) - P_{m,\widetilde{X}_g}(I_h))^2}{P_{m,\widetilde{X}_g}(I_h)}. \quad 12$$

This statistic was assumed to follow a chi-squared distribution with $\kappa = l - 3$ degrees of freedom [17] (the number of frequencies, l, reduced by the number of parameters of the fitted distribution, μ and σ, and the constraint $\sum_h P_{m,X_g}(I_h) = 1$). This gave the p-value, which is the probability of observing a test statistic *at least* as extreme, i.e. 1 minus the cumulative distribution function of χ_g^2 for κ degrees of freedom. Finally, our Gaussian-fit model was deemed adequate for a given feature in a group $\mathscr{L}_g$ if its p-value was greater than 0.05 (Supplementary Fig. 5).

Note that the chi-squared test of goodness-of-fit produces inaccurate results if the size of the sample is too small—i.e., by convention, less than 5 elements in a bin [17]. Reducing the number of frequencies l increases the number of elements in each bin, but also decreases the number of degrees of freedom *kappa*, which must remain greater than 3 for the test to be applicable here. Based on these constraints, 128 distributions out of 252 had to be excluded from the evaluation.

For most of the remaining 124 distributions, the validity of our assumptions about their type (normal or log-normal) could be retained. Only 20 of them had a p-value under 0.05, among which 12 were under 0.01

(Supplementary Fig. 5). The complete ranges of p-values obtained were the following: [3.7e-8,0.42] for cell cycle lengths x_i, [3.6e-6,0.46] for mitosis times m_i, [8.5e-3,0.89] for average cell volumes $\overline{v}_i$, [5e-3,0.96] for average cell surface areas $\overline{s}_i$, [0.06,0.84] for average volume ratios a_i, and [1.3e-3,0.76] for average surface area ratios b_i. The histogram of all 124 p-values is plotted in Supplementary Fig. 6.

Ideally, the worst-fit cases could be used to subdivide cell groups $\mathscr{L}_g$ into smaller classes (e.g., oral and aboral cells) reflecting the multimodal aspect of certain distributions, or to adopt different parametric models (e.g., exponential instead of Gaussian curves). Given the small size of the cohort, however, and the non-reproducibility of these outlier distributions in various specimens, it was difficult to rely on such deviations to define new cell types or new models. Moreover, biological relevance of the inferred cell clusters would have to be validated by correlation with the fate map and/or the gene expression in the form of a gene expression "atlas" [18], which is out of the scope of this study. This is why we preferred keeping the simplicity of normal and log-normal approximations as they captured the essential characteristics of statistical distributions (their mean and variance) even in marginal cases. For now, we left out any potential additional information for the sake of understandability and interpretability of the whole dataset. The description of distribution shapes can be refined in future work by using a larger cohort of specimens.

Supplementary Note 2.2.2 Cell volume and surface area dynamics

The volume and surface area of a cell are not constant during its cell cycle length because of various contractions and expansions. To highlight the fluctuations of the cellular geometry around its average, we define two other cell quantities:

- the *normalized volume*: $\omega_i(u) = v_i(t)/\overline{v}_i$
- the *normalized surface*: $\phi_i(u) = s_i(t)/\overline{s}_i$

where $u \in [0,1]$ represents the cell's "normalized age", defined as the percentage of elapsed cell cycle length:

$$t = m_j + ux_i = m_i - (1-u)x_i \;\Leftrightarrow\; u = \frac{t-m_j}{x_i} = \frac{t-m_j}{m_i - m_j} \qquad 13$$

(m_j is the mother's mitosis time). From there, the *microdynamics* of cell geometry can be described by taking the empirical mean of these temporally realigned quantities within each group $\mathscr{L}_g$ (Supplementary Figs. 7,8):

$$\widetilde{\omega}_g(u) = \frac{1}{N_g}\sum_{i\in\mathscr{L}_g}\omega_i(u) \;\text{ and }\; \widetilde{\phi}_g(u) = \frac{1}{N_g}\sum_{i\in\mathscr{L}_g}\phi_i(u), \qquad 14$$

making the assumption that the functions ω_g and ϕ_g are essentially deterministic, i.e. the variations in volume and surface *rescaled in amplitude and time* are the same for all cells $i \in \mathscr{L}_g$ (hence the notations $\widetilde{\omega}_g$, $\widetilde{\phi}_g$ instead of $\widetilde{\mu}_{\Omega_g}, \widetilde{\mu}_{\Phi_g}$). Compared to the other six empirical means calculated previously (equation 11), the particularity of these values is their dependency on time, which is rescaled to align the signals.

At the level of individual cells, by definition of a_i and b_i, the following relationships hold:

$$v_i(t) = \overline{v}_i\omega_g(u) = a_i\overline{v}_j.\omega_g(u) \qquad 15$$

$$s_i(t) = \overline{s}_i\phi_g(u) = b_i\overline{s}_j.\phi_g(u). \qquad 16$$

These equations mean that the volume and surface area variations can be decomposed into a stochastic and a deterministic component. The stochastic component, $a_i\overline{v}_j$ or $b_i\overline{s}_j$, concerns the evolution of the average volume or surface area along the cell lineage, while the deterministic component, ω_g or ϕ_g, modulates the cell volume or surface area around its average. The domain of these functions is the interval $[0,1]$,

representing the percentage u of elapsed cell cycle (their value is 0 outside this interval), and their range is comprised in $[0.8, 1.3]$. Using equations 5,6, their integral is also normalized:

$$1 = \frac{1}{x_i} \int_{m_i - x_i}^{m_i} \frac{v_i(t)}{\overline{v}_i} dt = \int_0^1 \omega_i(u) du \quad \Rightarrow \quad \int_0^1 \omega_g = 1 \tag{17}$$

$$1 = \frac{1}{x_i} \int_{m_i - x_i}^{m_i} \frac{s_i(t)}{\overline{s}_i} dt = \int_0^1 \phi_i(u) du \quad \Rightarrow \quad \int_0^1 \phi_g = 1. \tag{18}$$

Supplementary Note 2.2.3 Independence along the lineage

Having defined parametric representations $(\widetilde{\mu}, \widetilde{\sigma})$ for each cell feature distribution within each cell group $\mathscr{L}_g$, we wanted to investigate the *relationships* among these variables. Since cells are genealogically related, it was natural to hypothesize some degree of correlation between cells belonging to the same descent. With the goal to find a reduced number of parameters describing the phenomenology of the process, we assumed *linear dependencies* between the cell distributions of daughters and mothers, and among sisters. This is written $Y = \lambda + \nu X$, where the scalar parameters λ and ν can be estimated by linear regression based on a set of N realizations of the random variables X and Y: $\{(x_i, y_i)\}_{i=1,\ldots,N}$. The empirical optimal values $\widetilde{\lambda}$ and $\widetilde{\nu}$ are the ones that minimize the *residual sum of squares* over the samples:

$$SS_{\text{res}} = \sum_{i=1}^{N} (y_i - (\widetilde{\lambda} + \widetilde{\nu} x_i))^2 = \sum_{i=1}^{N} \varepsilon_i^2, \tag{19}$$

where ε_i denotes the residual error terms such that $y_i = \widetilde{\lambda} + \widetilde{\nu} x_i + \varepsilon_i$ and is assumed to be normally distributed around zero. Then, to characterize the accuracy of this linear estimation, we used the *coefficient of determination*, R^2, which measures the percentage of variation of one variable explained linearly by the other [19, 16]. If we define the *total sum of squares* by

$$SS_{\text{tot}} = \sum_{i=1}^{N} (y_i - \widetilde{y})^2 \quad \text{with} \quad \widetilde{y} = \frac{1}{N} \sum_{i=1}^{N} y_i, \tag{20}$$

where $\widetilde{y}$ is the empirical mean of Y, then the percentage of variation that remains unexplained linearly is given by the ratio $SS_{\text{res}}/SS_{\text{tot}}$, and the coefficient of determination is equal to its complement:

$$R^2 = 1 - \frac{SS_{\text{res}}}{SS_{\text{tot}}}. \tag{21}$$

For a "simple" linear regression (i.e. one with a single explanatory variable), it can be shown that the coefficient of determination is equal to the square of the estimated Pearson's coefficient of correlation:

$$R^2 = \widetilde{\rho}_{X,Y}^2, \quad \text{where} \quad \rho_{X,Y} = \frac{\text{cov}(X,Y)}{\sigma_X \sigma_Y} = \frac{\mu_{XY} - \mu_X \mu_Y}{\sigma_X \sigma_Y}. \tag{22}$$

The greater R^2, the more X and Y tend to be linearly dependent, with a binary decision threshold generally set at 0.6. Note that R^2 does not measure nonlinear dependencies, thus can remain low even if X and Y are strongly related through quadratic or logarithmic functions, for example.

Here, this coefficient was applied to six pairs of features calculated among the 252 distributions deemed sufficiently represented in the dataset (Supplementary Note 2.2.1, Supplementary Fig. 3). In the following, for a group index $g = (e, n, k)$ we use the shorthand notation $g - 1 = (e, n - 1, k)$ to refer to the previous-generation cell groups of the same type in the same embryo.

Daugther's cell cycle length vs. mother's mitosis time As above, we assumed a simple model of linear dependency between the cell cycle length x_i of a cell $i \in \mathscr{L}_g$ and the mitosis time m_j of its mother $j \in \mathscr{L}_{g-1}$: $x_i = \lambda + \nu m_j$. The coefficients of determination $R^2 = \widetilde{\rho}^2_{M_{g-1},X_g}$ were computed between the distributions $X_g \sim \{x_i \mid i \in \mathscr{L}_g\}$ and $M_{g-1} \sim \{m_j \mid j \in \mathscr{L}_{g-1}\}$ at several generations n. For this calculation, we used the 23 groups $\mathscr{L}_g$ of Supplementary Fig. 3a in which all cell cycles were completely known, i.e. had observed start and end mitosis times, and their corresponding 23 groups $\mathscr{L}_{g-1}$. In the end, the values of R^2 that we obtained fell in the range [3e-3,0.51], which indicated only weak or inexistent linear dependencies.

Sisters' cell cycle lengths For each pair of sister cells $i_1, i_2 \in \mathscr{L}_g$, ordered such that $x_{i_1} > x_{i_2}$, we also assumed $x_{i_1} = \lambda + \nu x_{i_2}$, where the longer cell cycle length was included in a set $X_{g,1}$ and the shorter one in another set $X_{g,2}$. The coefficient of determination $R^2 = \widetilde{\rho}^2_{X_{g,1},X_{g,2}}$ was then computed in the same 23 groups and the values obtained belonged to the interval [0.095,0.77], revealing some linear dependency among sisters' cell cycle lengths within certain groups. More precisely, R^2 coefficients scored above 0.6 in the following seven groups $g = (e,n,k)$: (4,7,Mac), (5,7,Mac), (3,8,Mac), (4,8,Mac), (1,7,Mes), (3,7,Mes) and (3,9,Mes). In the other 16 groups, there was no clear linear dependency.

Volume and surface area Concerning the volume and surface area, several models of dependency can be explored. Usually, it is assumed that the volume of a mother is conserved in its two daughters through mitosis, and when volumes are measured immediately prior to division and after division, this relationship can even be used to assess segmentation accuracy [6]. In addition, as generations 6 to 10 are part of cleavage stages, the global cellular volume is expected to remain constant while individual cell volumes are decreasing by half at each generation, as confirmed on average by Supplementary Figs. 3, 4C. Thus we may expect average volumes to verify: $\overline{v}_{i_1} + \overline{v}_{i_2} = \overline{v}_j$. Dividing by $\overline{v}_j$, this is equivalent to a linear relationship $a_{i_1} = 1 - a_{i_2}$ between the average daugther/mother volume ratios of the sister cells. To verify if there was such a dependency, we computed the coefficient of determination $R^2 = \widetilde{\rho}^2_{A_{g,1},A_{g,2}}$ as above between pairs of sister cells $i_1, i_2 \in \mathscr{L}_g$ ordered such that $a_{i_1} > a_{i_2}$. Over the 38 cell groups $\mathscr{L}_g$ of Supplementary Fig. 3c in which all volumes could be confidently measured, the coefficients of determination belonged to [1.4e-3,0.88], with 34 values below 0.61 and the other four values in [0.75,0.88] corresponding to cell groups (3,7,SMic), (3,7,Mac), (4,8,Mac) and (1,7,Mes). However, the $\widetilde{\lambda}$ and $\widetilde{\nu}$ parameters for these groups were found in $[-0.45,0.12]$ and $[0.87,3.02]$ respectively, i.e. far from 1 and -1. Altogether, these results contradicted the hypothesis of average cell volume conservation across mitosis through consecutive generations, as we observed a variation in the range of $\pm 20\%$ around 0.5 for the average daughter/mother volume ratio.

Daughter's volume (or surface area) ratio vs. mother's volume (or surface area) We investigated whether daughter cells compensated for the mother's deviation from the average either in terms of volume or in terms of surface area. Such compensation would mean that the average daughter/mother volume ratio a_i of a cell $i \in \mathscr{L}_g$ and the average volume $\overline{v}_j$ of its mother $j \in \mathscr{L}_{g-1}$ were correlated. However, the coefficients of determination R^2 computed in the same 38 groups as above ranged in [1.9e-4,0.43], indicating no linear relationship. The same tests were performed on surface area features, yielding similar negative results. All relationships are summarized in Table 3 and the histogram of all the coefficients of determination evaluated is shown on Supplementary Fig. 9.

However, although variability in volume and surface area at the individual cell level was not counterbalanced across consecutive generations, global uniformization could be observed at the macroscopic level, as shown in Fig. 3k (main article) for the evolution of the total cellular volume.

Overall, given the weak values of R^2 obtained for the above six features of individual features in the different

cell groups (R^2 was greater than 0.6 in only 20 cases out of 198 investigated pairs of distributions), we adopted the viewpoint that the various random variables were independent between and within cell groups. Although some of these correlation values may be ascribed to underlying deterministic factors responsible for cell-to-cell variability [20], our goal in the present work was to find parameters that could summarize relationships between random variables, hence the poor statistical significance of the linear regression test led us to a global assumption of independence. The following section examines the relations between probability laws in the different cell groups and how they are combined in the cell lineage.

Supplementary Note 2.3 Multi-level probabilistic model

In this stage, we used the parameterized description of the individual feature distributions (cell cycle length, mitosis time, volume, surface area) obtained above in each cell group, along with the assumption of independence among the different groups, to design a probabilistic model. As explained below, our model accurately predicted the final distributions of individual cell features compared to inter-individual differences within the cohort. It reproduced the embryo-level dynamics in each specimen (number of cells, total cellular volume, total cellular surface area), and also accounted for the propagation of intra-individual variability along the cell lineage.

Supplementary Note 2.3.1 Proliferation dynamics

In the first step toward designing a probabilistic model of the dynamics and evolution of the cell lineage, based on our data analysis, we stated the following relationships between the mitosis times and cell cycle lengths:

$$\begin{cases} M_g = M_{g-1} + X_g \\ M_g \sim \mathcal{N}(\mu_{M_g}, \sigma_{M_g}) \\ X_g \sim \mathcal{N}(\mu_{X_g}, \sigma_{X_g}) \\ P(X_g \mid M_{g-1}) = P(X_g) \end{cases} \qquad 23$$

where $\mathcal{N}(\mu, \sigma)$ represents a normal distribution of mean μ and variance σ^2 [16]. These equations implement the assumption of independence between X_g and M_{g-1}. They are also realized independently in each branch of the cell lineage by the usual relationship $m_i = m_j + x_i$ (where cell $j \in \mathcal{L}_{g-1}$ is the mother of cell $i \in \mathcal{L}_g$). From there, we could relate the parameters governing these distributions as follows:

$$\begin{cases} \mu_{M_g} = \mu_{M_{g'}} + \sum\limits_{h=g'+1}^{g} \mu_{X_h} \\ \sigma_{M_g} = \sqrt{(\sigma_{M_{g'}})^2 + \sum\limits_{h=g'+1}^{g} (\sigma_{X_h})^2} \end{cases} \qquad 24$$

where $g' = (e, n', k)$ denotes the "initial" group, i.e. $n' < n$ is the first generation at which cells of type k can be observed in embryo e, and we use the shorthand notation $g' + 1 = (e, n' + 1, k)$. The last equation means that the variability of mitosis times, represented by the variance of their Gaussian distribution, is based on the sum of variabilities of cycle lengths in each preceding cell group along the lineage tree. Similarly, the mean mitosis time is the sum of mean cell cycle lengths over the preceding groups.

Proof. Equation 24 can be derived from equation 23 by developing the recurrence relation between the

random variables:

$$\begin{aligned} M_g &= M_{g-1} + X_g \\ &= M_{g-2} + X_{g-1} + X_g \\ &= M_{g'} + X_{g'+1} + ... + X_{g-1} + X_g \end{aligned} \tag{25}$$

Recall that the observation window of the developing embryo in our data is limited. It begins at the earliest at the 32-cell stage, i.e. at the 6th cell cycle ($n' = 6$) where cells can be found in each of the four subpopulations, and ends at the latest at the 408-cell stage, i.e. cycle $n = 10$ for Mes and Mac cells, cycle $n = 9$ for LMic cells, and cycle $n = 7$ for SMic cells. Since the recurrence could not be traced all the way back to the origin, we provided a boundary condition for the initial distribution of mitosis times in the form of a Gaussian distribution, as in equation 23: $M_{g'} \sim \mathcal{N}(\mu_{M_{g'}}, \sigma_{M_{g'}})$. Then, the probability distribution of mitosis time M_g was derived from the property that the density of the sum of two independent random variables is equal to the convolution of their individual densities:

$$\begin{aligned} f_{M_g} = f_{M_{g-1}+X_g} &= f_{M_{g-1}} * f_{X_g} \\ &= f_{M_{g-2}} * f_{X_{g-1}} * f_{X_g} \\ &= f_{M_{g'}} * f_{X_{g'+1}} * ... * f_{X_{g-1}} * f_{X_g} \end{aligned} \tag{26}$$

where f_Y denotes the probability density of Y and $*$ is the convolution product. In the case of Gaussian distributions, the convolution of two distributions is another Gaussian whose mean and variance are the sums of their respective components [21]:

$$f_Y = f(y \mid \mu, \sigma) = \frac{1}{\sigma\sqrt{2\pi}} e^{-\frac{(y-\mu)^2}{2\sigma^2}} \tag{27}$$

$$f_{Y_1} * f_{Y_2} = f(y \mid \mu_1, \sigma_1) * f(y \mid \mu_2, \sigma_2) = f\left(y \mid \mu_1 + \mu_2, \sqrt{\sigma_1^2 + \sigma_2^2}\right) \tag{28}$$

Since each distribution of cell cycle lengths is a Gaussian distribution, $f_{X_g} = f(x \mid \mu_{X_g}, \sigma_{X_g})$ and $f_{M_{g'}} = f(m \mid \mu_{M_{g'}}, \sigma_{M_{g'}})$, we obtained equation 24 by combining equations 26 and 28:

$$f_{M_g} = f\left(m \mid \mu_{M_{g'}} + \sum_{g'+1}^{g} \mu_{X_h}, \sqrt{(\sigma_{M_{g'}})^2 + \sum_{g'+1}^{g} (\sigma_{X_h})^2}\right) \tag{29}$$

This result guarantees that the knowledge of the distributions of cell cycle lengths in each cell group was sufficient to characterize the distributions of mitosis times. □

Supplementary Note 2.3.2 Cell volume and surface area dynamics

In a second step, we modelled the stochastic component of the volume variations as follows (Supplementary Note 2.2.2):

$$\begin{cases} \overline{V}_g = \overline{V}_{g-1} A_g \\ \overline{V}_g \sim \log \mathcal{N}(\mu_{\overline{V}_g}, \sigma_{\overline{V}_g}) \\ A_g \sim \log \mathcal{N}(\mu_{A_g}, \sigma_{A_g}) \\ P(A_g \mid \overline{V}_{g-1}) = P(A_g) \end{cases} \tag{30}$$

where $Y \sim \log \mathcal{N}(\mu, \sigma)$ denotes a log-normal distribution, i.e. $\log(Y)$ is normally distributed with mean μ and variance σ^2 [22, 16]. As above, these equations implement the assumption of independence between

A_g and $\overline{V}_{g-1}$ and are realized independently by $\overline{v}_i = \overline{v}_j a_i$. From there, we also obtained:

$$\begin{cases} \mu_{\overline{V}_g} = \mu_{\overline{V}_{g'}} + \sum_{h=g'+1}^{g} \mu_{A_h} \\ \sigma_{\overline{V}_g} = \sqrt{(\sigma_{\overline{V}_{g'}})^2 + \sum_{h=g'+1}^{g} (\sigma_{A_h})^2} \end{cases} \qquad 31$$

by a proof similar to the previous section, with the difference that we were dealing here with log-normal distributions, hence the recurrence relationship was:

$$\overline{V}_g = \overline{V}_{g'} \left(\prod_{h=g'+1}^{g} A_h \right) \Leftrightarrow \log(\overline{V}_g) = \log(\overline{V}_{g'}) + \sum_{h=g'+1}^{g} \log(A_h). \qquad 32$$

Finally, to obtain v_i from $\overline{v}_i$, we used equation 15 with a deterministic function ω_g (the "microdynamics" model of the variation of volume occurring between two consecutive mitoses) estimated from the empirical curves of Supplementary Fig. 7. Note that there is no simple probabilistic model for V_g directly, only for $\overline{V}_g$.

All the above equations are the same for surface areas, replacing $\overline{V}_g$, A_g, v_i and ω_g with $\overline{S}_g$, B_g, s_i and ϕ_g, respectively.

Supplementary Note 2.3.3 Model summary

The model is summarized in Supplementary Table 4, which describes the relationships between random variables at the level of cell groups, and Supplementary Table 5, which recapitulates how the probabilistic variables are instantiated in each branch of the lineage tree. Note that, just like there was no easy analytical model for the non-renormalized volume and surface area random variables, V_g and S_g, it is also difficult or impossible to give an analytical expression for the probability distributions of the embryo-level quantities (defined in Supplementary Note 2.1.2 and measured in Supplementary Note 2.1.3): number of cells $N^k(t)$, total cellular volume $W^k(t)$, and total cellular surface area $Z^k(t)$. Although these quantities are themselves random variables, there is no simple theoretical model for the *macroscopic dynamics* of cell populations. They could be assessed, however, by numerical simulation via the generation of virtual cell lineages, as explained in Supplementary Note 2.3.5 below.

Supplementary Note 2.3.4 Model evaluation

To assess the relevance of our analytical model, we evaluated the difference between, on the one hand, the empirical distributions (M_g, $\overline{V}_g$, $\overline{S}_g$) measured directly on the embryos and, on the other hand, their counterparts (M_g^*, $\overline{V}_g^*$, $\overline{S}_g^*$) predicted by the model from the first group g' and the sums of (X_h, A_h, B_h) over the cycles. This was done in each "final" group $g = g'' = (e, n'', k)$, i.e. where n'' is the last generation at which the random variable can be observed on a sufficient number of cells of type k in embryo e, and should also verify $n'' > n'$. For 5 embryos, 3 variables and 4 types, there is a total of 60 potential final groups, but in practice only 48 satisfied this condition and were retained.

To this aim, we relied on the "Kullback-Leibler (KL) divergence", which measures the discrepancy between two probability densities p and q [23, 24]:

$$D_{\mathrm{KL}}(p \,||\, q) = \int p(x) \log \frac{p(x)}{q(x)} dx. \qquad 33$$

With Gaussian distributions $p = \mathcal{N}(x \mid \mu_p, \sigma_p^2)$ and $q = \mathcal{N}(x \mid \mu_q, \sigma_q^2)$, the KL divergence becomes:

$$D_{\mathrm{KL}}(p \,||\, q) = -\log \frac{\sigma_p}{\sigma_q} + \frac{1}{2} \left(\frac{\sigma_p^2}{\sigma_q^2} + \frac{(\mu_p - \mu_q)^2}{\sigma_q^2} - 1 \right) \qquad 34$$

and can be symmetrized into a "distance" as follows:

$$\Delta_{\text{KL}}(p,q) = \frac{1}{2}\Big(D_{\text{KL}}(p\,||\,q) + D_{\text{KL}}(q\,||\,p)\Big) \tag{35}$$

$$= \frac{1}{4}\left(\frac{\sigma_p^2}{\sigma_q^2} + \frac{\sigma_q^2}{\sigma_p^2} + \Big(\frac{1}{\sigma_p^2} + \frac{1}{\sigma_q^2}\Big)(\mu_p - \mu_q)^2 - 2\right) \tag{36}$$

Therefore, we calculated $\Delta_{\text{KL}}(M_g, M_g^*)$ by plugging in the above formula the following parameter values:

$$\begin{cases} \mu_p = \mu_{M_g} \quad \text{and} \quad \mu_q = \mu_{M_g^*} = \mu_{M_{g'}} + \sum\limits_{h=g'+1}^{g} \mu_{X_h} \\ \sigma_p = \sigma_{M_g} \quad \text{and} \quad \sigma_q = \sigma_{M_g^*} = \sqrt{(\sigma_{M_{g'}})^2 + \sum\limits_{h=g'+1}^{g} (\sigma_{X_h})^2} \end{cases} \tag{37}$$

where $g = g''$, and same thing for $\Delta_{\text{KL}}(\overline{V}_g, \overline{V}_g^*)$ and $\Delta_{\text{KL}}(\overline{S}_g, \overline{S}_g^*)$.

An absolute distance, however, cannot be interpreted if it is not compared to other typical interindividual distances within the cluster of groups. This is why we replaced Δ_{KL} with a normalized version $\widehat{\Delta}_{\text{KL}}$ calculated *relatively* to each embryo of the cohort, for example in the case of M_g in specimen 1:

$$\widehat{\Delta}_{\text{KL}}(M_{g(1)}, M_{g(1)}^*) = \frac{\Delta_{\text{KL}}(M_{g(1)}, M_{g(1)}^*)}{\frac{1}{|\mathscr{E}|-1} \sum\limits_{e \in \mathscr{E}, e \neq 1} \Delta_{\text{KL}}(M_{g(1)}, M_{g(e)})} \tag{38}$$

where $g(1) = (1,n,k)$ and $g(e) = (e,n,k)$. These normalized distances between the model and the data were computed for division times, volumes and surface areas, in each cell population k and relatively to each embryo e that verified $n'' > n'$, i.e. displayed at least one measurable cycle. Results are summarized in the histogram of Supplementary Fig. 10. Among the 48 eligible groups, 75% showed a close proximity ($\widehat{\Delta}_{\text{KL}} < 0.5$ in 36 cases) between measured and predicted parameter sets, 21% were still in good agreement ($0.5 \leq \widehat{\Delta}_{\text{KL}} < 1.5$ in 10 cases), and 4% fell far away ($\widehat{\Delta}_{\text{KL}} > 3$ for $\overline{V}$ and $\overline{S}$ in the Mes cells of embryo 5). Therefore, except for these two outliers, our probabilistic model accurately predicted the dynamics of sea urchin development.

Supplementary Note 2.3.5 Simulation of artificial cell lineages

Based on the above model, realistic virtual cell lineages were generated and their statistics compared to the real data. Starting from the 32-cell stage (16 Mes, 8 Mac, 4 LMic, 4 SMic cells), a cell lineage was computed by letting each cell divide into two and implementing the relationships between microscopic features (Supplementary Table 5), based on the empirical and estimated values of the parameters (Supplementary Note 2.2.1). This used the temporally and spatially rescaled datasets of the specimens according to the coefficients calculated previously (Supplementary Note 2.1.3).

For each embryo, 300 realizations of the cell lineage were produced by varying the random generator's seed. As Supplementary Fig. 1 illustrates, all cell lineages shared the same binary tree topology (if taking into account only the bifurcation nodes), whereas branches could be of variable lengths (along the temporal axis) depending on mitosis times. Another difference between embryos resided in their respective windows of observation, i.e. the specific intervals $[n', n'']$ during which there were enough observable cells of type k.

Comparison between simulated specimens and empirical values is shown in Supplementary Fig. 11 on three macrosopic quantities: total number of cells $N(t)$, total cellular volume $W(t)$, and total cellular surface area $Z(t)$. Given the finite window of observation, the last cell cycles were often incomplete. As a consequence, the volume and surface area microdynamics, ω_g and ϕ_g (representing the deterministic "carrier waves" in equations 15,16) could not be correctly estimated (shaded areas).

Supplementary Note 2.4 Prototype

In sum, our probabilistic model at the coarse-grained level of cell groups $\mathscr{L}_g$ provides an invariant framework relating individual cell features to embryo-level dynamics. The whole cohort can be described by the same system of equations (based on normal distributions), while cell statistics allows us to extract specific parameter values for each variable of interest in each group.

In this final step, we wanted to obtain a unified representation, or *prototype*, of the sea urchin blastula's normal development. To this aim, individual measurements were aggregated over the embryo index e using the geometrical concept of *centroid* of a set of points, which generalizes the notion of average in high-dimensional spaces such as probability distributions. The mathematical field of "information geometry" is especially relevant to treat these spaces, in particular equip them with a distance such as the "Bregman divergence" or the KL divergence [23, 25]. Here, we kept with our use of the latter to compute centroid distributions for each generation-type pair (n,k). For example, in the case of variable X:

$$X_{g(0)} = \arg\min_X \left(\frac{1}{|\mathscr{E}|} \sum_{e\in\mathscr{E}} \Delta_{\mathrm{KL}}(X, X_{g(e)}) \right), \tag{39}$$

where $g(0) = (0,n,k)$ and 0 denotes the prototype's index. Extending the assumption of normal (or log-normal) distributions to the prototype, we write $X_{g(0)} \sim \mathscr{N}(\mu_{X_{g(0)}}, \sigma_{X_{g(0)}})$, and therefore the prototype's mean and variance parameters can be found by solving the following minimization problem:

$$(\mu_{X_{g(0)}}, \sigma_{X_{g(0)}}) = \arg\min_{(\mu,\sigma)} \left(\frac{1}{4|\mathscr{E}|} \sum_{e\in\mathscr{E}} \frac{\sigma^2}{\sigma^2_{X_g}} + \frac{\sigma^2_{X_g}}{\sigma^2} + \left(\frac{1}{\sigma^2} + \frac{1}{\sigma^2_{X_g}}\right)(\mu - \mu_{X_g})^2 - 2 \right). \tag{40}$$

Numerical results for the usual six variables, based on their empirical parameters in each cell group $\mathscr{L}_g$, are plotted in Supplementary Fig. 3. In addition, the prototypical volume and surface area microdynamics, which are deterministic functions, are simply obtained by averaging the group-specific variables over the cohort (Supplementary Figs. 7 and 8, in bold):

$$\widetilde{\omega}_{g(0)}(u) = \langle \widetilde{\omega}_g(u) \rangle_{e\in\mathscr{E}} = \frac{1}{|\mathscr{E}|} \sum_{e\in\mathscr{E}} \widetilde{\omega}_g(u) \tag{41}$$

$$\widetilde{\phi}_{g(0)}(u) = \langle \widetilde{\phi}_g(u) \rangle_{e\in\mathscr{E}} = \frac{1}{|\mathscr{E}|} \sum_{e\in\mathscr{E}} \widetilde{\phi}_g(u). \tag{42}$$

Finally, as it was done for each embryo, 300 realizations of the cell lineage were also produced for the prototype by varying the random generator's seed, then compared with the empirical values (Fig. 3j-l).

Supplementary Note 3 Spatial modelling

Based on the above digital reconstruction and probabilistic model, the last stage of our study consisted of a spatially explicit computational model and simulation of the sea urchin embryo, based on the MecaGen platform [26],[17].

Supplementary Note 3.1 Biomechanical model

Supplementary Note 3.1.1 Equation of motion

Newton's second law states that the acceleration $\vec{a}$ of a body is proportional to the sum of the forces $\vec{F}$ applied to it. The proportionality factor is the inverse of the mass of the body, a quantity assumed to remain constant over time: $\vec{a} = \vec{F}/m$. In particle-based physics, for each particle characterized by a mass m, acceleration $\vec{a}$, location $\vec{X}$, velocity $\vec{v}$, and radius R, forces are a function of the last three quantities, and Newton's law reads

$$m\vec{a} = \vec{F}(\vec{X}, \vec{v}, R). \tag{43}$$

At the scale of the biochemical world, however, this classical formulation does not reflect the emergent and rather counterintuitive phenomena that cells exhibit individually and collectively. Cells are so small and their interactions so "sticky" that the physics of their motion is radically different from the one to which we are accustomed at our scale. The main difference resides in the disappearance of inertial forces [27] and its consequence is that in a "low Reynolds number" environment, applied forces produce a velocity, not an acceleration. Therefore, displacement is proportional to instantaneous forces and inversely proportional to a damping coefficient λ:

$$\vec{v} = \vec{F}/\lambda \tag{44}$$

Generalizing to a multicellular system, the motion of each cell indexed by i is governed by its interactions with all other cells j belonging to its neighbourhood denoted by $\mathcal{N}_i$, according to

$$\lambda_i \vec{v}_i = \sum_{j \in \mathcal{N}_i} \vec{F}_{ij} \tag{45}$$

Supplementary Note 3.1.2 Cell properties

Each cell is represented by a single cylindrical particle, i.e. its relaxed shape is considered isotropic in the surface plane. The cylinder axis $\vec{U}$ is orthogonal to the epithelial surface. The radius R of the cylinder delimits the distance occupied by each cell in the surface plane, and its half-height denoted by $R^{\perp}$ characterizes the depth of the tissue. Throughout this study, we assume $R^{\perp}$ homogeneous and constant across the embryo: for each cell i and at any time t, $R_i^{\perp}(t) = R_0^{\perp}$. Therefore, the relation between a cell's current radius and volume $V_i(t)$ reads:

$$R_i(t) = \sqrt{\frac{V_i(t)}{2\pi R_0^{\perp}}} \tag{46}$$

Supplementary Note 3.1.3 Spatial neighbourhood

A cell sustains forces exerted by the neighbouring cells in contact with it. At any point in time, the embryo is described by a set of cylindrical particles i, each of them characterized by a location $\vec{X}_i = (x_i, y_i, z_i)$, a radius R_i, a half-height $R_i^{\perp}$ and an axis $\vec{U}_i$. We need to infer from these intrinsic parameters the *neighbourhood links* between cells. To this goal, we followed a two-step strategy: first, a preselection of potential neighbours that obey certain metric criteria, then a refinement of this list according to topological features. After that, we specify how the cell axis is updated from the new list.

Metric selection Two cells of radius R_i, R_j and locations $\vec{X}_i$, $\vec{X}_j$ are considered neighbours according to a metric criterion if and only if their relative distance is less than the sum of their radii $R_i + R_j$ multiplied by a constant factor $c_{\max}$. Thus the metric neighbourhood of cell i is defined as follows:

$$\mathcal{N}_i^m = \left\{ j \in S : \left\| \vec{X}_i - \vec{X}_j \right\| \leq c_{\max}(R_i + R_j) \right\} \quad 47$$

Denoting the distance between the two cells by r_{ij} and the cutoff value by $r_{ij}^{\max}$, it means that j is a metric neighbour of i if and only if $r_{ij} \leq r_{ij}^{\max}$. The radius $r_{ij}^{\max}$ sets the maximum distance of the cell deformation in the surface plane, i.e. the spheroidal domain of space inside which cells can potentially interact. We assume that this maximum distance is reached when cells deform by a factor $c_{\max} = 2.0$.

Topological selection A purely metric neighbourhood is not viable in our multicellular context as it often leads to cell volumes collapsing during simulation when the adhesion between interacting cells is high. This is why we filter the set $\mathcal{N}_i^m$ via a topological criteria to obtain a reduced set $\mathcal{N}_i^t$. To do this, a 3D Delaunay triangulation could be employed but it is computationally too slow because it involves heavy data structures composed of multiple tetrahedra. Our own solution is inspired by a similar neighbourhood model, the *Gabriel graph*. In 2D, whereas the Delaunay triangulation imposes that no node be found inside the circumcircle of any triangle, the Gabriel graph method requires that no node be found inside the circle whose diameter is a valid neighbourhood edge.

Here, we propose to generalize the Gabriel criterion to the 3D case directly and add a parameter to control the size of the circle: two cells (i, j) are considered neighbours if no other cell k of the embryo is located inside the sphere whose origin is the centre of segment $\overline{ij}$, which is $(\vec{X}_i + \vec{X}_j)/2$, and whose diameter is $\alpha_{\text{gab}} \|\vec{X}_i - \vec{X}_j\|$, where :

$$\mathcal{N}_i^t = \left\{ j \in \mathcal{N}_i^m : \forall k \in \mathcal{N}_i^m, \left\| \vec{X}_k - \frac{1}{2}(\vec{X}_i + \vec{X}_j) \right\| \geq \frac{\alpha_{\text{gab}}}{2} \left\| \vec{X}_i - \vec{X}_j \right\| \right\} \quad 48$$

To our knowledge, the term Gabriel graph has not been formally defined in 3D. However, when it happens to be mentioned [28], triangles are used instead of segments to define circumspheres. This makes sense, as in n dimensions, the Delaunay triangulation uses n-simplices for its basic units (triangles in 2D, tetrahedra in 3D) while the Gabriel graph uses $(n-1)$-simplices (edges in 2D, triangles in 3D). Here, however, we will continue using edges in 3D while still referring to our method as a Gabriel graph. Note that in the 2D case, the Gabriel graph is a subgraph of the Delaunay triangulation. Every pair of neighbours in a Gabriel graph is also a pair of neighbours according to the Delaunay criteria.

Cell axis Once the topological list of neighbours is determined, the surface orientation of the monolayered sea urchin can be expressed at each cell location by averaging the outward normal vectors of the n triangles formed by n topological neighbours (Fig. 4a). These triangles are obtained by sorting the list of neighbours in counterclockwise order around the current axis $\vec{U}_i$. Let's denote by a_k with $k = 0...n-1$ the neighbour indices in the sorted list. The surrounding triangles are $X_i X_{a_k} X_{a_{k+1}}$ with $k = 0...n-2$ and $X_i X_{a_{n-1}} X_{a_0}$. The updated cell axis is calculated by summing the surrounding outward vector axes:

$$\vec{U}_i \leftarrow \sum_{k=0}^{n-2} \frac{(\vec{X}_{a_k} - \vec{X}_i) \times (\vec{X}_{a_{k+1}} - \vec{X}_i)}{\|(\vec{X}_{a_k} - \vec{X}_i) \times (\vec{X}_{a_k+1} - \vec{X}_i)\|} + \frac{(\vec{X}_{a_{n-1}} - \vec{X}_i) \times (\vec{X}_{a_0} - \vec{X}_i)}{\|(\vec{X}_{a_{n-1}} - \vec{X}_i) \times (\vec{X}_{a_0} - \vec{X}_i)\|} \quad 49$$

The cell axis is then normalized:

$$\vec{U}_i \leftarrow \frac{\vec{U}_i}{\|\vec{U}_i\|} \quad 50$$

Supplementary Note 3.1.4 Forces

The interaction force $\vec{F}_{ij}$ exerted by cell j over cell i leads the swarm of cells toward an equilibrium state. It is the sum of two components:

- an *attraction-repulsion* interaction force $\vec{F}_{ij}^{\parallel}$, which maintains the integrity of the cell's volume and controls both the stiffness and the adhesion of the interaction in the direction of the surface plane, and
- a *planarity conservation* interaction force $\vec{F}_{ij}^{\perp}$, which maintains the planarity or "monolayeredness" of the epithelium; this force can be modulated via a planar rigidity coefficient.

Moreover, the damping coefficient λ_i is proportional to the surface of the cylindrical cell: $\lambda_i = \lambda_0 S_i$ with $S_i = 2\pi R_i(R_i + 2R_i^{\perp})$. Therefore, the equation of motion used in this model becomes:

$$\vec{v}_i = \frac{1}{\lambda_0 S_i} \sum_{j \in \mathcal{N}_i} \left(\vec{F}_{ij}^{\parallel} + \vec{F}_{ij}^{\perp} \right) \qquad 51$$

Attraction-repulsion force This force is derived from an attractive/repulsive interaction potential between two neighbouring cells i, j, and it is colinear with the neighbourhood vector $\vec{u}_{ij} = (\vec{X}_i - \vec{X}_j)/\|\vec{X}_i - \vec{X}_j\|$ (here, from j to i). The rationale of this potential is to *maximize the contact surface area* between any two neighbours in the swarm. For every pair of neighbouring cells (i, j) we focus on the attraction-repulsion interaction potential $E_{ij}^{\parallel}$ from which $\vec{F}_{ij}^{\parallel}$ is derived, and model it from three juxtaposed domains:

- a *repulsion* domain (decreasing E) at distances shorter than a certain equilibrium distance defined by $r_{ij}^{\mathrm{eq}} = c_i^{\mathrm{eq}} R_i + c_j^{\mathrm{eq}} R_j$, where in the most general case c_i^{eq} is a coefficient that can vary from cell to cell
- an *attraction* domain (increasing E) at distances greater than r_{ij}^{eq}
- a *neutral* domain (constant E) beyond the maximum limit of the interaction field $r_{ij}^{\max} = c_{\max}(R_i + R_j)$

Coefficient c_i^{eq} is established explicitly in this study by considering that the equilibrium state of the swarm is reached when the cells form the densest packing in the 2D plane, i.e. the hexagonal lattice if disks are identical. The ratio of the surface occupied by the disks to the surface containing the disks is equal to the ratio of the surface of a disk to the surface of an hexagon (its Voronoi domain), i.e. $\pi/(2\sqrt{3}) \simeq 0.9068997....$ Therefore, in the perfect uniform arrangement here, the equilibrium distance between any cells (i, j) of equal radii $R_i = R_j = R$ is constant and reads

$$r_{ij}^{\mathrm{eq}} = 2c_{\mathrm{eq}} R, \text{ with } c_{\mathrm{eq}} = \left(\frac{\pi}{2\sqrt{3}} \right)^{1/2} \simeq 0.9523128... \qquad 52$$

We now generalize the notion of equilibrium distance to pairs of unequally sized neighbour cells. In this case, the common radius of equilibrium depends on individual contributions from each cell, which is simply proportional to their radius, keeping the same constant coefficient c_{eq} as above:

$$r_{ij}^{\mathrm{eq}} = c_{\mathrm{eq}} R_i + c_{\mathrm{eq}} R_j = c_{\mathrm{eq}}(R_i + R_j) \qquad 53$$

Given these boundary conditions, we consider here the simplest form of schematic mechanical model that can realize both the short-range repulsion and long-range attraction parts, namely: *spring-like forces derived from an elastic potential*. It means that forces $\vec{F}^{\parallel}$ are a linear function, and potentials $E^{\parallel}$ a quadratic function of $(r - r_{ij}^{\mathrm{eq}})$.

Mechanical interactions between neighbouring cells have been extensively studied in biophysics, yet

due the multiprotein and polymeric nature of the cytoskeleton, together with the membrane's flexibility and adhesiveness, it remains an extremely elusive topic and difficult to summarize by a definitive set of equations. Roughly, one consensus hypothesis is that a major driving mechanism can be characterized by intercellular *surface tension*. The two main components of this tension are cellular *adhesion*, which tends to lower it, and cellular *cortical tension*, which acts antagonistically. The balance between these two components determines the ultimate behavior of the interaction, i.e. attractive or repulsive.

Another fundamental mechanism must also be taken into account: *volume preservation* in cells. As we made the assumption that cells are cylindrical and cell interactions *in the plane* can be decoupled into two individual, additive contributions, we assume here that the volume conservation principle plays a role in the *repulsive* part of the potential only. This allow us to introduce a correction in the form of a discontinuity in the force derivative at the distance of equilibrium r_{ij}^{eq}. We will also globally refer as "adhesion" to the cumulated effect of true surface adhesion and its antagonist, cortical tension.

Accordingly, to modulate the intensity of adhesion, the expression of $\vec{F}^{\parallel}$ is split into two parts, one below and one above the equilibrium distance r_{ij}^{eq}, corresponding to two different stiffness coefficients w. A low (resp. high) adhesion coefficient w will induce a weak (resp. strong) attraction:

$$\vec{F}_{ij}^{\parallel,\text{lin}} = \begin{cases} -w_{\text{rep}}(r_{ij} - r_{ij}^{\text{eq}}).\vec{u}_{ij} & \text{if } r_{ij} < r_{ij}^{\text{eq}} \\ -w_{\text{adh}}(r_{ij} - r_{ij}^{\text{eq}}).\vec{u}_{ij} & \text{if } r_{ij} \geq r_{ij}^{\text{eq}} \end{cases} \tag{54}$$

Cells i, j start to adhere only when their relative distance becomes less than $r_{ij}^{\max}$. As the intensity of the attraction can not be suddenly maximal at that point, a half-bell shape between r_{ij}^{eq} and $r_{ij}^{\max}$ is better suited to model a more realistic cellular interaction. We obtain this shape by multiplying the linear force $\vec{F}_{ij}^{\parallel,\text{lin}}$ by a term $A(r_{ij}, R_i, R_j)$ scaling as the area of the surface between i and j. We decided to use a monomial of degree 2 to make it scale like a surface. If the distance between two double-size neighbouring cells is doubled, the area of the surface of contact should quadruple:

$$A(r_{ij}, R_i, R_j) = \begin{cases} \left(r_{ij} - r_{ij}^{\max}\right)^2 & \text{if } r_{ij} < r_{ij}^{\max} \\ 0 & \text{if } r_{ij} \geq r_{ij}^{\max} \end{cases}, \text{ where } r_{ij}^{\max} = c_{\max}(R_i + R_j) \tag{55}$$

In the whole interval $[0, r_{ij}^{\max}]$, the depth of the well is controlled by a repulsion coefficient w_{rep} (until r_{ij}^{eq}) and an adhesion coefficient w_{adh} (beyond r_{ij}^{eq}; see Fig. 4b). Thus the final expression of the nonlinear version of the relaxation force that takes into account the contact surface area reads:

$$\vec{F}_{ij}^{\parallel} = A_{ij}.\vec{F}_{ij}^{\parallel,\text{lin}} = \begin{cases} -w_{\text{rep}}(r_{ij} - r_{ij}^{\max})^2(r_{ij} - r_{ij}^{\text{eq}}).\vec{u}_{ij} & \text{if } r_{ij} < r_{ij}^{\text{eq}} \\ -w_{\text{adh}}(r_{ij} - r_{ij}^{\max})^2(r_{ij} - r_{ij}^{\text{eq}}).\vec{u}_{ij} & \text{if } r_{ij} \geq r_{ij}^{\text{eq}} \text{ and } r_{ij} < r_{ij}^{\max} \\ \vec{0} & \text{if } r_{ij} \geq r_{ij}^{\max} \end{cases} \tag{56}$$

Finally, to compare our custom attraction/repulsion force $\vec{F}_{ij}^{\parallel}$ with classical potentials, we show their superimposed plots in Supplementary Fig. 13.

Planar rigidity force Attraction-repulsion forces are not sufficient to ensure that the spatial configuration of the embryo will remain locally "planar", i.e. monolayered, during simulation, spherical. Indeed, such forces are colinear to the neighbourhood vectors $\vec{u}_{ij}$, causing the epithelium to eventually agglomerate into a multilayered tissue. To model a monolayered epithelial sheet, we introduce a specific *planar rigidity force* (Supplementary Fig. 14), which aims at maintaining each neighbour cell in the lateral region, orthogonally to the normal vector. Between two neighbour cells i and j, this force is aligned with the bisector of their normal vectors: $\vec{n}_{ij} = (\vec{U}_i + \vec{U}_j)/\|\vec{U}_i + \vec{U}_j\|$, and is proportional to their distance, the dot product between $\vec{u}_{ij}$ and $\vec{n}_{ij}$, and a planar rigidity coefficient, k_{rig}:

$$\vec{F}_{ij}^{\perp} = -k_{\text{rig}} r_{ij} (\vec{u}_{ij}.\vec{n}_{ij}) \vec{n}_{ij} \tag{57}$$

Supplementary Note 3.1.5 Mitoses

Mitoses are triggered when the cell cycle is complete, i.e. when the amount of time since the last mitosis is equal to the randomly generated cell cycle length (Supplementary Note 2.3.5). When that event occurs, a division axis $\vec{M}_m$ is randomly selected in the local tangential plane of the tissue. Based on the dividing cell's normal axis $\vec{U}_m$, the local tangential plane is defined by a couple of orthonormal vectors $(\vec{U}'_m, \vec{U}''_m)$ as follows:

$$\begin{aligned} \vec{U}'_m &= \frac{1}{\sqrt{(U_m^x)^2+(U_m^y)^2}}(-U_m^y, U_m^x, 0) \\ \vec{U}''_m &= \vec{U}_m \times \vec{U}'_m \end{aligned} \qquad 58$$

The random division axis $\vec{M}_m$ is generated in the tangential plane by drawing an angle α uniformly from $[0, 2\pi]$ and setting:

$$\vec{M}_m = \cos(\alpha)\vec{U}'_m + \sin(\alpha)\vec{U}''_m \qquad 59$$

As mentioned in Supplementary Note 3.1.2, the apicobasal radii of the daughter cells is assumed equal to their mother's: $R^{\perp}_{d_1} = R^{\perp}_{d_2} = R^{\perp}_m = R^{\perp}_0$, and can be inferred from their respective volume. The daughters are positioned at a distance proportional to the mother's radius along the division axis:

$$\begin{aligned} \vec{X}_{d_1} &= \vec{X}_m + 0.15 R_m \vec{M}_m \\ \vec{X}_{d_2} &= \vec{X}_m - 0.15 R_m \vec{M}_m \end{aligned} \qquad 60$$

an their axes are identical to the mother's: $\vec{U}_{d_1} = \vec{U}_{d_2} = \vec{U}_m$.

Supplementary Note 3.2 Comparison to experimental data

Supplementary Note 3.1 described how we combined the prototypical cell lineage, derived from the data, and the biomechanical model, which includes several free parameters. The values of these free parameters leading to the most realistic spatial embedding of the prototypical cell lineage can be obtained by finding the best fit between the simulations and the original data. To this end, we designed a metric based on the topology of the cells in the embryo (D_s) and two objective functions characterizing certain properties of the simulated embryos only (S_s and P_s; see below). The resulting parameter space exploration is described in Supplementary Note 3.2.3.

Supplementary Note 3.2.1 Metric

The topology of the cells in an embryo can be represented by the network of cell-cell contacts [11]. The vertices of the network are the nuclei and the edges of the network are the junctions between cells. For any cell $i \in \mathscr{L}$ at a given time t, we denote by $\mathscr{N}_i(t)$ its neighbourhood, defined as the set of cells that are one edge away from i. With this, the number of neighbours that a cell of a given population k shares within another population l is the basis for the definition of the border between these two populations at a time t:

$$\mathscr{B}_{k\to l}(t) = \left\{ i \in \mathscr{L}^k(t) : \exists j \in \mathscr{N}_i(t), \text{ such that } j \in \mathscr{L}^l(t) \right\} \qquad 61$$

and the number of neighbours of a cell i at this border:

$$b_i^{k\to l}(t) = \left| \{ j \in \mathscr{N}_i(t) \text{ such that } j \in \mathscr{L}^l(t) \} \right| \qquad 62$$

To evaluate a spatial simulation, we compared the characteristics of its borders with that of the measured embryo.

Distance between distributions Given two empirical probability distributions p and q, considered on the bins X, such that $\sum_{x \in X} p(x) = 1$, we define the following measure of similarity [29]:

$$d(p,q) = \frac{1}{2} \sum_{x \in X} \left(\sqrt{p(x)} - \sqrt{q(x)} \right)^2 \quad 63$$

which is always positive, equal to 1 when the two distributions do not overlap and 0 when they are equal.

Histogram resampling To overcome the problem of having different time samplings in the various embryos, we used a common time interval across all specimens and for the spatial simulation, ranging from 240 min post fertilization (mpf) to 702 mpf, with a sample every 3 min. We interpolated the value of the histograms used for the comparison at each time point of this time interval.

Metric We used the *distribution* over i of the numbers of neighbours $b_i^{k \to l}$ that a cell i shares at a border to compare simulations and digitally reconstruct empirical embryos using the distance defined above, averaged over the different time points, populations, and embryos. The comparison protocol is described in algorithm 1, where $h_e^{k \to l}(t)$ and $h_s^{k \to l}(t)$ denote the distributions of $b_i^{k \to l}(t)$ in an embryo e and a simulation s respectively. It leads to a metric D_s whose value is comprised between 0 (best fit) and 1 (worst fit).

```
Given a simulated embryo s:
for each specimen e of the cohort do
    for each ordered pair (k → l) of populations do
        for each time t of the common time interval do
            compute the distance between the two distributions:
            d_{e,s}^{k→l}(t) = d(h_e^{k→l}(t), h_s^{k→l}(t))
        end
        compute the average over the entire time interval:
        D_{e,s}^{k→l} = ⟨d_{e,s}^{k→l}(t)⟩_t
    end
    compute the average over all population pairs in e:
    D_{e,s} = ⟨D_{e,s}^{k→l}⟩_{k→l}
end
compute the average over the whole cohort:
D_s = ⟨D_{e,s}⟩_{e∈ℰ}
```

Algorithm 1: Comparison protocol between simulations and measured embryos

Supplementary Note 3.2.2 Objective functions

In addition to the metric introduced above, we measured intrinsic features of the simulated embryos via objective functions. These functions are defined for a given time step and subsequently averaged over the entire time interval.

Sphericity objective function S_s An important criteria to evaluate the model is the shape of the simulated embryos. For that purpose, we designed S_s to be the ratio of the standard deviation over the average of the set of distances between the cell positions $\vec{X}_i$ and the embryo centre $\vec{X}_0 = \langle \vec{X}_i \rangle_i$. This function has a low

value for a small standard deviation and a large average, and is null in the case of a spherical embryo:

$$S_s = \frac{\sigma}{\mu}, \quad \text{where} \quad \mu = \frac{1}{|\mathscr{L}|}\sum_{i\in\mathscr{L}} \|\vec{X}_i - \vec{X}_0\| \quad \text{and} \quad \sigma = \sqrt{\frac{1}{|\mathscr{L}|}\sum_{i\in\mathscr{L}} \left(\|\vec{X}_i - \vec{X}_0\| - \mu\right)^2} \qquad 64$$

Planarity objective function P_s For each cell, this function characterizes the unicity of the epithelium layer by measuring the ratio of cells belonging to the surrounding apicobasal neighbourhood. The neighbourhood $\mathscr{N}_i$ is split into two complementary sets, one covering the lateral domain $\mathscr{N}_i^{\|}$, the other the apical domain $\mathscr{N}_i^{\perp}$:

$$\mathscr{N}_i^{\|} = \left\{ j \in \mathscr{N}_i : -\vec{u}_{ij} \cdot \vec{U}_i < \eta \right\} \qquad 65$$

$$\mathscr{N}_i^{\perp} = \left\{ j \in \mathscr{N}_i : -\vec{u}_{ij} \cdot \vec{U}_i \geq \eta \right\} \qquad 66$$

where η is a threshold value controlling the partition between the two sets, here $\eta = \cos(\pi/4)$. The planarity objective function is then written:

$$P_s = \frac{1}{|\mathscr{L}|}\sum_{i\in\mathscr{L}} \frac{|\mathscr{N}_i^{\perp}|}{|\mathscr{N}_i|} \qquad 67$$

For a monolayered epithelium, $P_s = 0$, otherwise its value increases as cells agglomerate into a 3D tissue.

Supplementary Note 3.2.3 Validation in parameter space

The purpose of this exploration study is to identify the parameter sets that produce a realistic spatial folding of the sea urchin embryo during its development. The validation criteria are the sphericity of the global embryo shape, measured by S_s, the maintenance of the monolayered epithelium, measured by P_s, and the similarity of the interpopulation border shapes, measured by D_s. Each criterion is explored separately and its optimal parametric region identified (main article, Fig. 4e-g). The common parameter space here is four-dimensional: it comprises the planar rigidity coefficient k_{rig}, the Gabriel criteria coefficient α_{gab} and the two adhesion coefficients specifically controlling the attractive part of the relaxation force $\vec{F}_{ij}^{\|}$: $w_{\text{adh,o}}$ between homotypic cells and $w_{\text{adh,e}}$ between heterotypic cells. The other free parameters of the model (namely w_{rep}, λ_0, and $c_{\max}$) are set to constant values (Supplementary Table 6). In Fig. 4 and Supplementary Videos 4-7, k_{rig} and α_{gab} are also set to constant values, while Fig. 4c-g shows only a restricted two-dimensional region of the explored domain in the ($w_{\text{adh,o}}$, $w_{\text{adh,e}}$) plane. Finally, the time increment Δt between two simulated time steps is 6 seconds.

Note concerning the force amplitude coefficients $w_{\text{adh,e}}$, $w_{\text{adh,o}}$, w_{rep} and k_{rig}: these coefficients are all divided by the damping coefficient λ_0 in the master equation of motion. Thus, simulations will be strictly equivalent if all these parameters are multiplied by the same factor.

For the spatial simulation, an initial spatial state of the cells was needed in order to begin the simulation at the 32-cell stage: we chose the measured initial state of one of the five embryos of the cohort.

Supplementary Software 1 Supplementary Software 1

The compressed folder ”prototype.zip” contains all the MATLAB files necessary to perform and visualize the complete statistical analysis of the cell lineage and to generate the prototype. It has been tested with Matlab R2011b and Matlab R2015a. A tutorial is provided in the file README.pdf contained in the folder.

References

[1] B. Rizzi and N. Peyriéras. Towards 3D in silico modeling of the sea urchin embryonic development. *Journal of chemical biology*, 7(1):17–28, 2014.

[2] E. Faure, T. Savy, B. Rizzi, C. Melani, M. Remešíková, R. Špir, O. Drblíková, D. Fabrges, M. Hammons, R. Čunderlík, G. Recher, B. Lombardot, L. Duloquin, I. Colin, J. Kollár, S. Desnoulez, P. Affaticati, B. Maury, A. Boyreau, J. Y. Nief, P. Calvat, P. Vernier, M. Frain, G. Lutfalla, Y. Kergosien, P. Suret, R. Doursat, A. Sarti, K. Mikula, N. Peyriéras, and P. Bourgine. A workflow to process 3D+time microscopy images of developing organisms and reconstruct their cell lineage. *Nature Communications*, 7(8674), 2016.

[3] A. Sarti, R. Malladi, and J. A. Sethian. Subjective surfaces: a method for completing missing boundaries. *Proceedings of the National Academy of Sciences*, 97(12):6258–6263, 2000.

[4] C. Zanella, M. Campana, B. Rizzi, C. Melani, G. Sanguinetti, P. Bourgine, K. Mikula, N. Peyriéras, and A. Sarti. Cells segmentation from 3D confocal images of early zebrafish embryogenesis. *IEEE Transactions on Image Processing*, 19(3):770–781, 2010.

[5] S. Kirkpatrick, C. D. Gelatt, and M. P. Vecchi. Optimization by simulated annealing. *Science*, 220(4598):671–680, 1983.

[6] N. Olivier, M. A. Luengo-Oroz, L. Duloquin, E. Faure, T. Savy, I. Veilleux, X. Solinas, D. Débarre, P. Bourgine, A. Santos, and N. Peyriéras. Cell lineage reconstruction of early zebrafish embryos using label-free nonlinear microscopy. *Science*, 329(5994):967–971, 2010.

[7] Z. Krivá, K. Mikula, N. Peyriéras, B. Rizzi, A. Sarti, and O. Stašová. 3D early embryogenesis image filtering by nonlinear partial differential equations. *Medical image analysis*, 14(4):510–526, 2010.

[8] S. Osher and J. A. Sethian. Fronts propagating with curvature-dependent speed: algorithms based on Hamilton-Jacobi formulations. *Journal of computational physics*, 79(1):12–49, 1988.

[9] L. C. Evans and J. Spruck. Motion of level sets by mean curvature I. *J. Diff. Geom*, 33(3):635–681, 1991.

[10] E. H. Davidson, R. A. Cameron, and A. Ransick. Specification of cell fate in the sea urchin embryo: summary and some proposed mechanisms. *Development*, 125(17):3269–3290, 1998.

[11] M. C. Gibson, A. B. Patel, R. Nagpal, and N. Perrimon. The emergence of geometric order in proliferating metazoan epithelia. *Nature*, 442(7106):1038–1041, 2006.

[12] L. M. Escudero, L.da F. Costa, A. Kicheva, J. Briscoe, M. Freeman, and M. M. Babu. Epithelial organisation revealed by a network of cellular contacts. *Nature communications*, 2:526, 2011.

[13] D. J. Aldous. *Exchangeability and related topics*. Springer, 1985.

[14] Y. S. Chow and H. Teicher. *Probability theory: independence, interchangeability, martingales*. Springer, 2003.

[15] P. Diaconis and D. Freedman. Finite exchangeable sequences. *The Annals of Probability*, 8(4):745–764, 1980.

[16] George Casella and Roger L Berger. *Statistical inference*, volume 2. Duxbury Pacific Grove, CA, 2002.

[17] J. H. McDonald. *Handbook of biological statistics*, volume 2. Sparky House Publishing Baltimore, MD, 2009.

[18] C. Castro-González, M. A. Luengo-Oroz, L. Duloquin, T. Savy, B. Rizzi, S. Desnoulez, R. Doursat, Y. L. Kergosien, M. J. Ledesma-Carbayo, P. Bourgine, N. Peyriéras, and A. Santos. A digital framework to build, visualize and analyze a gene expression atlas with cellular resolution in zebrafish early embryogenesis. *PLoS computational biology*, 10(6):e1003670, 2014.

[19] C. R. Rao. *Linear statistical inference and its applications*, volume 22. John Wiley & Sons, 2009.

[20] O. Sandler, S. P. Mizrahi, N. Weiss, O. Agam, I. Simon, and N. Q. Balaban. Lineage correlations of single cell division time as a probe of cell-cycle dynamics. *Nature*, 519(7544):468–471, 2015.

[21] P. A. Bromiley. Products and convolutions of Gaussian distributions. *Medical School, Univ. Manchester, Manchester, UK, Tech. Rep*, 3:2003, 2003.

[22] E. Limpert, W. A. Stahel, and M. Abbt. Log-normal distributions across the sciences: keys and clues. *BioScience*, 51(5):341–352, 2001.

[23] S. Amari and H. Nagaoka. *Methods of information geometry*, volume 191. American Mathematical Society, 2007.

[24] F. Nielsen and R. Nock. Sided and symmetrized Bregman centroids. *Information Theory, IEEE Transactions on*, 55(6):2882–2904, 2009.

[25] C. R. Rao. Information and accuracy attainable in the estimation of statistical parameters. *Bulletin of the Calcutta Mathematical Society*, 37(3):81–91, 1945.

[26] J. Delile, R. Doursat, and N. Peyriéras. Computational modeling and simulation of animal early embryogenesis with the MecaGen platform. In *Computational systems biology (2nd ed)*, pages 359–405. Elsevier, 2013.

[27] E. M. Purcell. Life at low Reynolds number. *Am. J. Phys*, 45(1):3–11, 1977.

[28] Dominique Attali and Annick Montanvert. Computing and simplifying 2D and 3D continuous skeletons. *Computer Vision and Image Understanding*, 67(3):261–273, 1997.

[29] Kameo Matusita. Decision rules, based on the distance, for problems of fit, two samples, and estimation. *The Annals of Mathematical Statistics*, pages 631–640, 1955.
